# LLPS droplet size estimation via UV–Vis spectroscopy using a microplate reader

**DOI:** 10.1038/s41598-025-33638-8

**Published:** 2025-12-27

**Authors:** Mayu Enomoto-Kusano, Takashi S. Kodama, Suai Anzawa, Kyoko Furuita, Ryoga Kobayashi, Naotaka Sekiyama, Wataru Togawa, Toshimichi Fujiwara, Yohei Miyanoiri, Hidehito Tochio, Chojiro Kojima

**Affiliations:** 1 Graduate School of Engineering Science, Yokohama National University, Tokiwadai 79-5, Hodogaya-ku, Yokohama, 240-8501 Japan; 2 Institute for Protein Research, The University of Osaka, Yamadaoka 3-2, Suita, 565-0871 Osaka Japan; 3 Department of Biophysics, Graduate School of Science, Kyoto University, Kitashirakawa Oiwake-cho, Sakyo-ku, Kyoto, 606-8502 Japan

**Keywords:** Biological techniques, Biophysics, Optics and photonics, Physics

## Abstract

Liquid-liquid phase separation (LLPS) is critical to the formation of membraneless organelles within cells. Monitoring the size and dynamics of LLPS droplets is essential to understanding their function. However, conventional methods, such as microscopy and dynamic light scattering (DLS), have limitations, including low throughput and complex setups. Here, we present a simple, label-free, high-throughput method for estimating droplet size using ultraviolet-visible (UV–Vis) spectra obtained using a standard microplate reader. This method is theoretically supported by Mie scattering theory. By analyzing the spectral shape and comparing it to that of glass beads of known sizes, our method enables the quantitative estimation of LLPS droplet size in a minimally invasive manner. We validated our approach using peptide droplets of known sizes, then applied it to monitor the growth of protein condensates formed by vesicle-associated membrane protein-associated protein B (VAPB). This technique offers a convenient, scalable alternative for screening LLPS dynamics in various biomolecular systems.

**Supplementary Information:**

The online version contains supplementary material available at 10.1038/s41598-025-33638-8.

## Introduction

Liquid-liquid phase separation (LLPS) is a phenomenon in which proteins form condensates with liquid-like properties. In cells, LLPS results in the formation of membrane-less organelles (MLOs), which play important roles in various biological processes, including transcription, translation, and stress response^1–3^.

Multivalent interactions drive spherical droplet formation by LLPS-associated proteins. The properties of these droplets are influenced by the interaction strength, type, and multivalency, as well as environmental conditions^4^. Therefore, accurate measurements of droplet dynamics and properties can help infer the underlying intermolecular interaction properties.

Various methods are known and used to study the dynamics and properties of LLPS droplets, such as ultraviolet-visible (UV‒Vis) spectroscopy, dynamic light scattering (DLS), atomic force microscopy (AFM), transmission electron microscopy (TEM), fluorescence recovery after photobleaching (FRAP), and fluorescence resonance energy transfer (FRET)^5^. UV‒Vis is useful for turbidity measurements of the droplets, DLS is useful for estimating the droplet size (diameter) distribution in solution, AFM helps in the study of droplet morphology and mechanical properties^6^, and TEM visualizes the internal structure of solutions at the nanoscale and provides detailed information on droplet morphological characteristics^7^. FRAP assesses the fluidity within the droplets and is valuable for studying the dynamics of proteins and RNA^8^, while FRET reveals the spatial structure and composition of droplets based on their fluorescence signal^9^. Each of these techniques offers unique advantages for studying different aspects of LLPS, such as droplet size, morphology, structure, and composition^10–12^.

Among the aforementioned LLPS detection methods, turbidity measurements have a strong advantage of being a simple method and intrinsically containing information on both the number and size of LLPS droplets. However, there is currently no simple method to quantitatively extract the size information from the turbidity measurements.

This study aimed to develop a simple, accessible, high-throughput method for estimating the size of LLPS droplets using UV‒Vis spectra acquired by a microplate reader. Theoretical investigation of Mie scattering shows that UV‒Vis spectral shape depends on the particle size. By focusing on the spectral shape derived from scattering and referencing standard particles of known sizes, we developed a quantitative assay that allows for the real-time monitoring of droplet dynamics, eliminating the need for specialized optics or labeling. The method was validated using peptide droplets of known sizes, and its application to vesicle-associated membrane protein-associated protein B (VAPB) droplets was demonstrated. This approach complements conventional microscopy and DLS, offering researchers a practical tool for studying LLPS, particularly in large-scale or time-course experiments.

## Theory

### UV‒Vis scattering theory

Depending on the particle size, light scattering by small particles in suspension is described by three theories^13^. For UV‒Vis scattering, when the particle size (diameter) is Φ50 nm or smaller, Rayleigh scattering theory is applied. When the particle size is larger than Φ50 nm, Mie scattering theory is applied. When the particle size is Φ100,000 nm or larger, geometrical optics theory is also applied for the sake of convenience in calculations.

### Mie scattering theory

Mie scattering theory is also known as Lorentz-Mie-Debye scattering theory. The notation used to describe Mie scattering theory has undergone considerable evolution over time. Significant discrepancies exist in the notation used in notable treatises^14–23^. In this paper, we follow the notation in Kerker’s book^21^, and the theory focuses on elastic scattering. It assumes that the incident and scattered light wavelengths are identical and does not consider inelastic scattering, such as Raman scattering. It also assumes incoherent single scattering under the following conditions: the particle concentration is sufficiently low, the average distance between particles is sufficiently large, and the particles are randomly positioned. For details of the theory, see the supplemental text and the references cited therein.

According to Bouguer’s law or the Lambert–Beer law, the attenuation of light passing through a scatterer layer is expressed as follows:

1 $$\:-\frac{dI}{dx}=N\:{C}_{\mathrm{e}\mathrm{x}\mathrm{t}}\:I$$

where *N* is the number of scatterer particles per unit volume, *C*_ext_ is the extinction cross section, and $\:I$ is the light intensity. The extinction cross section *C*_ext_ is the sum of the absorption cross section (*C*_abs_), which is due to light absorption by particles, and the scattering cross section (*C*_sca_), which is due to light scattering by particles,

2 $$C_{{{\mathrm{ext}}}} = C_{{{\mathrm{abs}}}} + C_{{{\mathrm{sca}}}}$$

Under conditions where scatterers’ light absorption can be negligible, *C*_ext_ is equal to *C*_sca_.

Mie scattering theory is derived under boundary conditions at the particle surface from the full formal solution to Maxwell’s equations including vector wave equations for incident light, light inside the particle, and scattered light^20,21^. The scattering property, such as *C*_sca_, is a function of the refractive indices of both the particle and the surrounding medium, the wavelength of light in the medium, and the particle size. The function under consideration is defined by an infinite series involving Legendre polynomials and Bessel functions, and it cannot be expressed in a simple form. The spectral shape obtained by measuring turbidity at various wavelengths is known as a fingerprint of the particle properties and is useful to estimate the particle size^22^.

### Concentration dependence

The transmittance *T*, which is the attenuation of light, can be obtained by integrating Eq. 1 as follows:

3 $$\:T=\frac{I}{{I}_{0}}=\mathrm{e}\mathrm{x}\mathrm{p}\left(-N\:{C}_{\mathrm{e}\mathrm{x}\mathrm{t}}\:l\right)=\mathrm{e}\mathrm{x}\mathrm{p}(-\tau\:\:l)$$

where $\:{I}_{0}$ is the incident light intensity, $\:l$ is the optical path length, and $\:\tau\:$ is the turbidity. The optical density (*OD*) can be obtained by taking the negative logarithm of Eq. 3 as follows:

4 $$\:OD=-\mathrm{log}\left(\frac{I}{{I}_{0}}\right)=\frac{\tau\:\:l}{\mathrm{l}\mathrm{n}10}=\frac{N\:{C}_{\mathrm{e}\mathrm{x}\mathrm{t}}\:l}{\mathrm{l}\mathrm{n}10}\approx\:\frac{N\:{C}_{\mathrm{e}\mathrm{x}\mathrm{t}}\:l}{2.3}$$

This indicates that *OD* value varies linearly with the number of particles per unit volume, i.e., particle concentration. The degree to which absorption and scattering attenuate the transmitted light varies with light wavelength. This results in a spectrum expressed in optical density (OD), which is similar to an absorption spectrum. In this study, this extinction spectrum is referred to as the UV‒Vis spectrum or the UV‒Vis scattering/turbidity spectrum.

### Additivity

Based on [Eq. 4], the *OD* value for a unit path length is additive for particles of each size when a mixture of particles of different sizes is suspended (i.e., a polydisperse system):

5 $$\:OD\approx\:\sum\:_{i}\frac{{N}_{\mathrm{i}}\:{C}_{\mathrm{e}\mathrm{x}\mathrm{t},\mathrm{i}}\:l}{2.3}=\frac{{N}_{\mathrm{t}}\:l}{2.3}\sum\:_{i}{C}_{\mathrm{e}\mathrm{x}\mathrm{t},\mathrm{i}}\:{f}_{\mathrm{i}}$$

where $\:{N}_{\mathrm{i}}$ and $\:{C}_{\mathrm{e}\mathrm{x}\mathrm{t},\mathrm{i}}$ are the number of particles per unit volume and the scattering cross section of the i-th size, respectively, and $\:{N}_{\mathrm{t}}$ and $\:{f}_{\mathrm{i}}$ are the total number of particles per unit volume and the fraction of particles of the i-th size, respectively. Since the observed *OD* value corresponds to the sum of the scattering contributions from all particles, the spectral shape represents a scattering intensity weighted average of the particle size distribution.

In Eq. 5, summation notation (∑) is used to account for the discrete nature of the particle size distribution. However, when the distribution is represented as a continuous function, the same property can be expressed in integral form^23^. In other words, the measured value for any particle size or concentration distribution corresponds to the integral of the scattering contributions from each infinitesimal layer along the optical path. Therefore, even if the particle concentration becomes nonuniform in the vertical direction due to sedimentation (i.e., a non-homogeneous system), the scattering observed in a vertical measurement system—through which light passes through all layers—corresponds to the sum of the scattering occurring in each layer.

## Results and discussion

### LLPS size detection method: scattering/turbidity measurement by UV‒Vis spectrum using a microplate reader

**Fig. 1:**
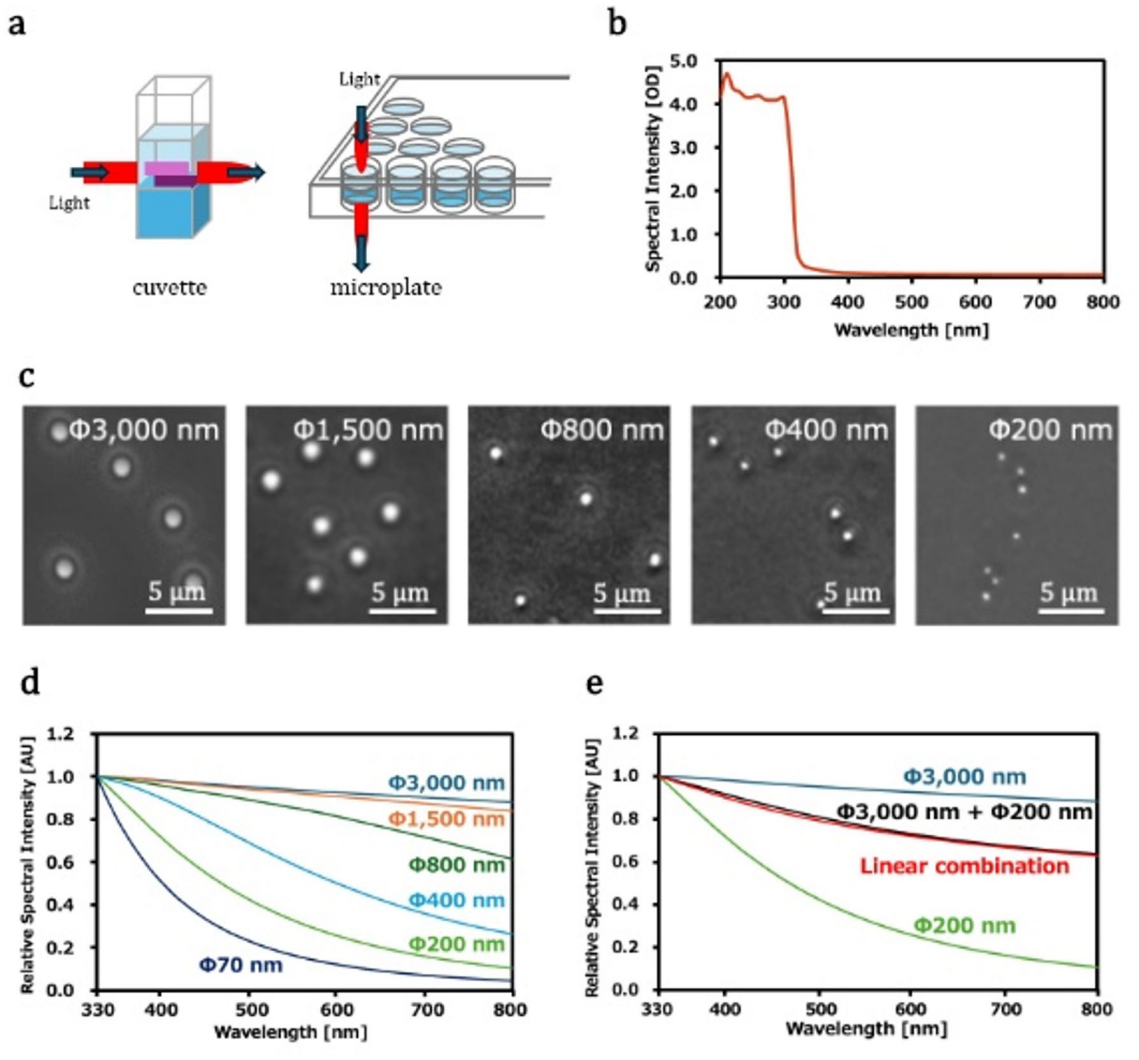
(**a**) Turbidity measurements using cuvette cells (left) and microplates (right). Two liquid phases are drawn as two boxes colored dark and light blue, respectively. The red lines represent the light transmission position. The turbidity value depends on the position of light transmission when cuvette cells are used but not when microplates are used. (**b**) UV‒Vis spectrum of the buffer without samples. (**c**) Microscopy images of glass beads with different particle sizes. (**d**) Spectral shapes of UV‒Vis spectra of glass beads with different particle sizes. (**e**) Additivity of the UV‒Vis spectra of mixture of two sizes of glass beads. (Black) The observed UV‒Vis spectra of 1:1 mixture of Φ3,000 nm and Φ200 nm of glass beads, and (Red) linear combination of each spectrum with weights of 1.467 and 0.536 for Φ3,000 nm and Φ200 nm, respectively.

Turbidity measurements using a cuvette cell are commonly used for LLPS monitoring. However, this method may have problems with quantitative detection. This is because the amount of LLPS formed at the top and bottom of the cuvette cell varies; the turbidity value varies depending on the light-transmission position (height). In contrast, turbidity measurements via microplates are suitable because light is transmitted vertically, and LLPS formation is robustly measured regardless of any variation in LLPS formation between the top and bottom of the microplate (Figs. 1a and S1). In addition, the microplate reader can consecutively measure multiple samples (up to 384 samples), and the sample required is less than 1/10 of the cuvette cells^24^.

First, the absorption characteristics of the microplate without samples were observed by measuring its UV‒Vis spectrum in the presence of the buffer solution. As shown in Fig. 1b, the polystyrene microplate exhibited intense UV absorption below 310 nm. Since the spectral intensity values beyond 4 cannot be quantitatively measured by the microplate reader we used, the detection wavelength should be above 310 nm, which is not hindered by the microplate absorption. For safety, it is preferable to be above 330 nm, where the spectral intensity value from the microplate was below 0.3, and the spectral intensity value from the sample below 3.7 is acceptable for quantitative measurements at 330 nm. Under this condition, the baseline correction quantitatively eliminates the residual absorbance of the microplate below 0.3.

UV absorption from colorless proteins typically occurs below 330 nm. Consequently, the light attenuation observed at wavelengths above 330 nm indicates that the samples are scattered light, rather than absorbing light. In this study, spectral intensities were measured as optical density (*OD*) values between 330 and 800 nm to detect LLPS droplets by UV‒Vis light scattering using the microplate reader.

### LLPS size detection method: particle size dependency evaluation using glass beads

The relationship between the shape of the UV‒Vis scattering/turbidity spectrum and the particle size of the glass beads was evaluated. The particle sizes of the used glass beads were Φ70, Φ200, Φ400, Φ800, Φ1,500, and Φ3,000 nm. These numbers are given in the manufacturer’s data sheet (Micromod particle technology GmbH, Germany) and were confirmed by microscopic observation, except for Φ70 nm (Fig. 1c). The size difference between Φ3,000 and Φ1,500 nm glass beads was not clear from microscopic observations (Fig. 1c, two on the left). This may be because Φ3,000 nm glass beads are too thick to neglect spherical aberration, which degrades image quality.

The UV‒Vis spectra of the glass beads of 6 different sizes revealed different spectral shapes (Fig. 1d). Larger particles, such as Φ3,000 and Φ1,500 nm glass beads, presented flatter spectra, and smaller particles, such as those with sizes of Φ200 and Φ70 nm, presented significantly greater decreases in spectral intensity versus wavelength.

Next, the additivity in spectral shape was evaluated by mixing glass beads of different sizes. The spectral shape was explained by a linear combination of the spectra for each glass bead when two glass beads of different sizes were mixed (Fig. 1e). These results support the idea that the LLPS size detection method, which uses the spectral shape of UV‒Vis spectrum, is an effective tool for evaluating particle size as a scattering intensity weighted average reflecting its distribution.

### Simulations of UV‒Vis scattering/turbidity spectra

**Fig. 2:**
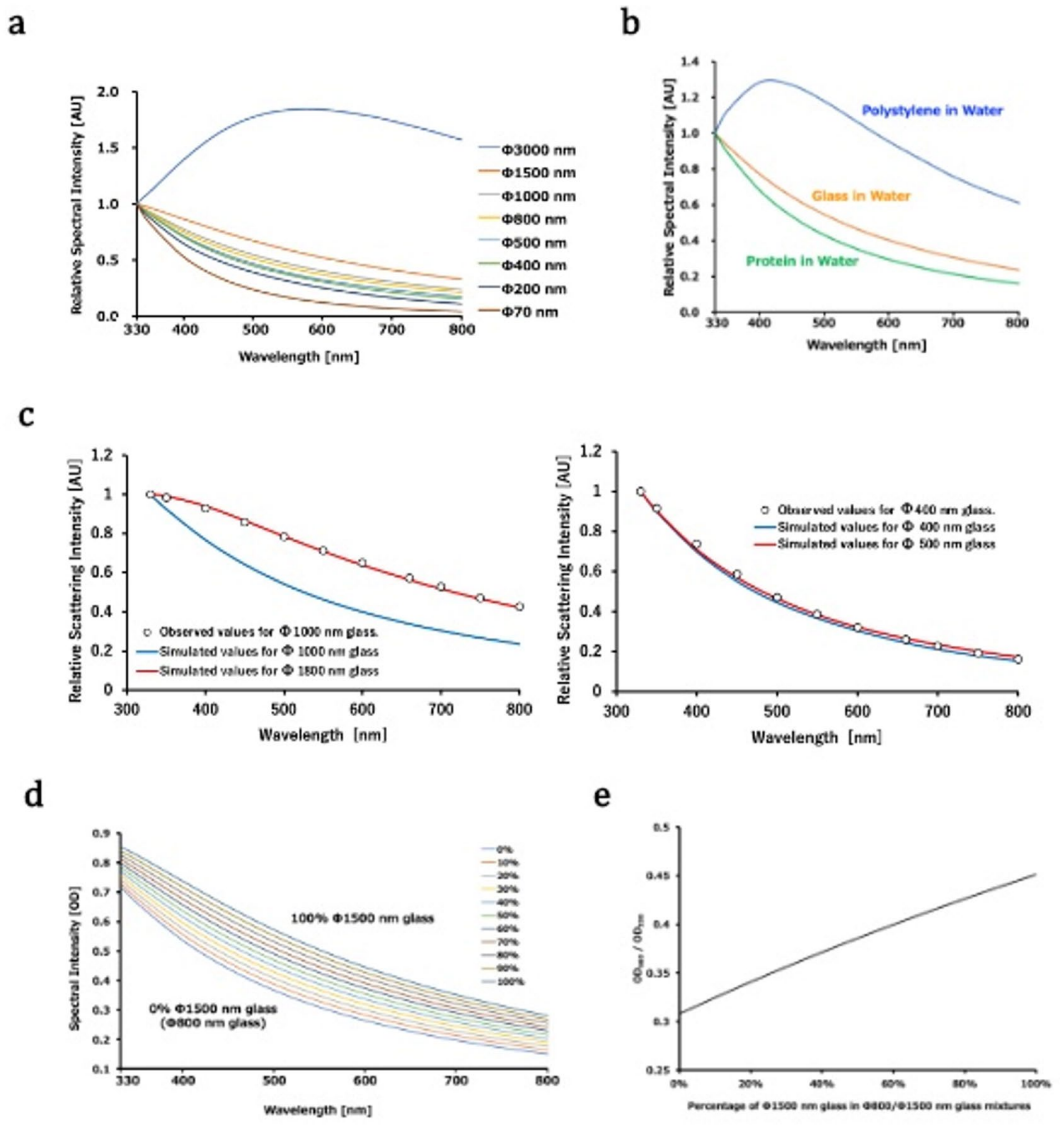
Simulated UV‒Vis scattering/turbidity spectra. (**a**) Spectral shapes for various-sized glass beads. (**b**) Same as (**a**), but for Φ800 nm particles composed of polystyrene (blue), glass (orange), and protein (green). (**c**) Observed spectral intensities (open circles) for Φ1,000 nm and Φ400 nm glass beads are shown in the left and right panels, respectively, along with the simulated spectra (blue lines). The best-fitted spectra, obtained by changing the particle sizes, are shown by the red lines. The experimental data for the Φ1,000 nm and Φ400 nm glass beads are from samples with a dilution factor of 0.0625 (Tables S3 and S6). (**d**) Simulated spectra with various percentages of Φ1,500 nm glass beads in a Φ800/Φ1,500 nm glass bead mixture while the total particle volume remains constant. This simulation is for the spectral change during the fusion process of small particles. (**e**) Same as (**d**) but showing the change in the *OD*_660_/*OD*_330_ value.

Based on Mie scattering theory, the UV‒Vis scattering/turbidity spectrum was numerically calculated assuming the glass beads were dispersed in water (Figure 2). The shape of the calculated spectrum (i.e., the wavelength dependence of turbidity) clearly depended on the size of the suspended particles (Figure 2a). This finding is consistent with the experimental results shown in Figure 1d.

Since the refractive indices of both the particle and the surrounding medium also affect the Mie scattering, the UV‒Vis scattering/turbidity spectra of particles of the same size with different refractive indices were calculated (Fig. 2b). Particles composed of polystyrene, glass, or protein solution dispersed in water have different refractive indices. The UV‒Vis scattering/turbidity spectrum of the polystyrene particles differs significantly from the others due to their relatively large refractive index, and this was experimentally supported (Figure S2). The calculated spectral shapes of the glass and protein particles are similar and gradually attenuate as the wavelength increases. As can be seen in Fig. 2a and b, the spectral shape of protein particles resembles that of smaller glass beads. However, this is also affected by the protein concentration inside and outside the particle, as well as the concentration of additives such as polyethylene glycol (PEG) (Figures S3). Therefore, these values should be interpreted as representing particle sizes equivalent to glass beads unless the exact refractive index inside and outside the particle is known.

### Relationship between the measured and simulated spectral shapes

The spectral shapes were calculated for various sizes and fitted to the shapes obtained experimentally for Φ1,000 and Φ500 nm glass beads (Fig. 2c). The best fit was obtained by assuming particle sizes of Φ1,800 and Φ400 nm in the calculation, respectively. In the region of larger particle sizes, the interpretation shifted significantly toward the larger particle sizes, although it remained within a factor of two. However, it should be noted that, overall, the calculated shapes explained the experimentally obtained spectral shapes well for both large and small particles.

### Dependence of the spectral intensity on the particle number and size

Theoretically, the scattering intensity is proportional to the particle number, but not the particle size. In simulations, the scattering intensity increases nonlinearly as the particle size increases for particles ranging from Φ35 to Φ3,000 nm (Figure S4). For example, the scattering intensity of Φ3,000 nm glass beads was over 10^7^ times higher than that of Φ35 nm glass beads. Therefore, the contribution of the smaller particles will be underestimated in the scattering analyses.

This nonlinearity complicates the evaluation of systems undergoing time-dependent changes, such as fusion and dissociation (e.g., LLPS). Consequently, the changes in particle number and size must be evaluated simultaneously. For example, when small droplets fuse to form larger droplets, the total number of particles in the system decreases proportionally to the cube of the particle size increase.

### Simulations of the spectral change during the fusion process of small particles

To examine how the spectrum changes in the fusion process, we calculated the UV‒Vis scattering/turbidity spectrum for a model mixture of Φ800 nm and Φ1,500 nm glass beads, assuming a gradual change in the ratio of the two (Fig. 2d, e). This simulation models the two-state transition process, whereby Φ800 nm droplets fuse to form Φ1,500 nm droplets.

Here, we consider that the volume of the Φ800 nm glass beads is (800/1,500)^3^ ~ 0.15 times that of the Φ1,500 nm glass beads, thereby reproducing a change in particle size distribution while the total particle volume remains constant. In LLPS, for example, this corresponds to the process in which many small particles gradually fuse together and are replaced by a small number of large particles at a constant total protein amount.

As all particles fuse, the total number of particles decreases by more than a factor of six. However, as the number of Φ1,500 nm particles increases due to fusion, the calculated scattering intensity increases across all wavelengths. This change in intensity is accompanied by a change in the spectral shape. Therefore, the shape of the UV‒Vis scattering/turbidity spectrum can, in principle, provide information about changes in particle size and the fraction of each component in the ensemble.

### The empirical approach to monitoring the fusion process

As shown in Figs. 1d and 2a, the difference in particle size affects the spectral shape, which can be calculated. However, calculating the spectral shape is a cumbersome process involving complicated steps, whether theoretical or numerical. About 100 years ago, the DQ method, an empirical method, was proposed to evaluate the size of suspended particles using the dispersion quotient (DQ) value^25^. The DQ value calculated from the turbidity at two wavelengths represents the spectral shape. Thus, the ratio of the spectral intensity at the short-wavelength side to the long-wavelength side (i.e., the DQ value) can be used to evaluate the particle size. In this report, the ratio of the *OD* values at 660 and 330 nm (*OD*_660_/*OD*_330_) is used to monitor changes in size quantitatively during the fusion process, as simulated in Fig. 2e.

### Validation of the LLPS size detection method using known-size model peptide droplets

The established LLPS size detection method was applied to model droplets consisting of a peptide fragment of the TIA-1 protein. TIA-1 was identified as an RNA-binding protein that plays a central role in the formation of stress granules by self-assembly via the C-terminal prion-like domain (PLD). TIA-1 has been shown to accumulate in pathological aggregates in brain tissue from mouse models of Huntington disease and tauopathy and in brain tissue from Alzheimer’s disease patients, where it co-aggregates with huntingtin and tau proteins^26–29^.

**Fig. 3:**
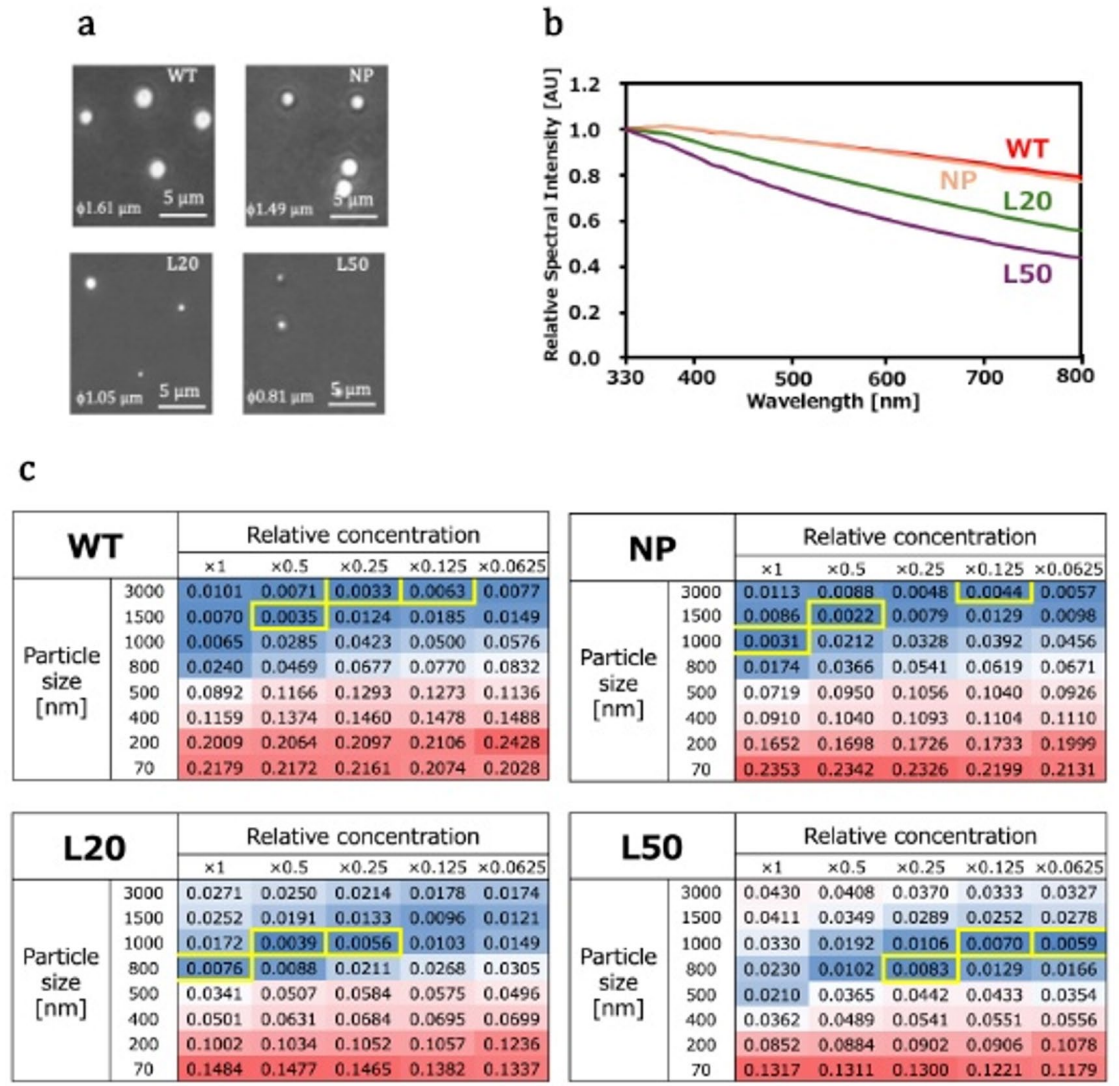
(**a**) Microscopy image of TIA-1 PLD-related peptide droplets (TIA-1 droplets). (**b**) The spectral shape of the TIA-1 droplets is shown by the relative spectral intensity, normalized to the *OD* value at 330 nm. (**c**) Heatmap showing the root mean square differences (RMSD) of the spectral shapes of the TIA-1 droplets compared to glass beads of known sizes. The standard concentration of each size of glass beads was adjusted so that the measured value at a wavelength of 330 nm was approximately 1.5, and then the beads were diluted in a series. The rows and columns represent the size (Φ3,000 nm to Φ70 nm) and relative concentration (×1 to ×0.0625), respectively, of the glass beads. The blue and red colors indicate small and large fitting residuals, respectively. The yellow squares represent the best-fit spectral shapes.

Kobayashi et al. developed a series of TIA-1 PLD-related peptides with different LLPS droplet sizes: WT, NP, L20, and L50 with median sizes of Φ1,550 nm, Φ1,450 nm, Φ940 nm, and Φ810 nm, respectively^30^. These peptides are designed to have different droplet formation properties while maintaining the same amino acid composition, and the refractive indices seem to be similar. Microscopic views of these TIA-1 droplets are shown in Fig. 3a. These droplets were evaluated via the microplate reader, as shown in Fig. 3b. The UV‒Vis spectra of WT and NP, which had similar particle sizes, were flatter and overlapped. Compared to WT and NP, L20 and L50, which have smaller particle sizes, showed greater decreases in spectral intensity versus wavelength. Therefore, the droplet sizes obtained by the microscopy are related to the UV‒Vis spectral shape obtained with the microplate reader.

### Estimation of TIA-1 droplet sizes by spectral shape fitting (heatmap method)

In theory, the shape of the normalized spectrum should not change with particle concentration in the case of single scattering. However, when measured with an actual device, the spectral shape is slightly affected by particle concentration. Furthermore, the experimental data and the simulated data diverge systematically for larger particles (Fig. 2c). This is thought to be due to instrumental non-ideality and the effects of multiple scattering in the sample, as described below. The degree of these effects depends on both the absolute amount of transmitted light and the anisotropy factor g of the particles. These effects cannot be avoided in situations of high particle concentration, low light transmittance, and large particle size (Figures S4-S6). In this context, when estimating the scattering intensity weighted average of the particle size from actual measurement data, spectral shape fitting to a measured standard spectrum that considers particle concentration is more appropriate than fitting to the simulated spectrum. Subsequent experiments adopted the spectral shape fitting method (heatmap method) based on measurement data for glass beads of known sizes (Tables S1-S8 and Figures S5-S6).

Figure 3c shows the results of fitting the spectra of glass beads of various sizes to the measured spectral shapes of droplets formed by four types of TIA-1 PLD-related peptides. The residuals from fitting the measured spectrum to the spectral shapes of glass beads of various sizes are a quantitative indicator of the assumed particle size. The heatmap display of the root mean square deviation (RMSD) obtained by fitting the measured data of glass bead suspensions of various concentrations and sizes provides an intuitive understanding of the estimated particle size of the sample.

Figure 3c clearly shows that the scattering intensity-weighted average particle sizes determined by the heatmap method for the four peptides (WT, NP, L20, and L50) are generally consistent with the average particle size observed by microscopy. The former sizes for WT, NP, L20, and L50 were Φ1,500–Φ3,000 nm, Φ1,000–Φ3,000 nm, Φ800–Φ1,000 nm, and Φ800–Φ1,000 nm, respectively, while the later sizes were Φ1,550 nm, Φ1,450 nm, Φ940 nm, and Φ810 nm, respectively. These results highlight the effectiveness of using the heatmap method to estimate the scattering intensity weighted average of the particle size.

### Application of the LLPS size detection method to protein droplets

Our LLPS size detection method was applied to the VAPB protein. The VAPB protein is an integral adaptor protein of the endoplasmic reticulum (ER) membrane that recruits a variety of interacting partners to the ER surface^31^. Through these interactions, the VAPB protein mediates a variety of processes, particularly the establishment of membrane contact sites between the ER and virtually all other cellular membranes. Previous research has shown that the P56S mutation of the VAPB protein is related to familial amyotrophic lateral sclerosis named ALS8^32–35^.

**Fig. 4:**
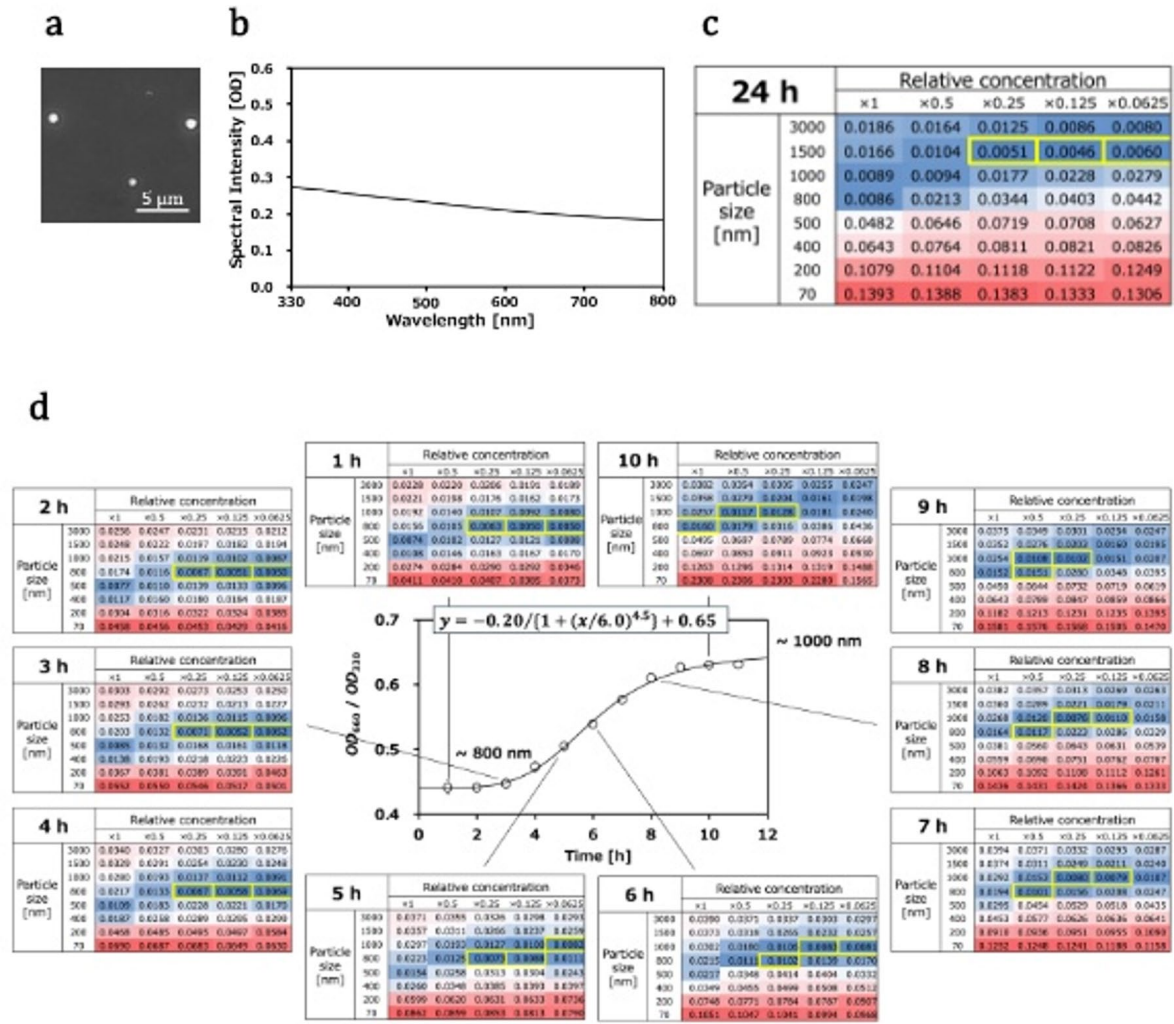
(**a**) Microscopy image of the VAPB droplets. (**b**) Spectral shape of VAPB LLPS is shown by spectral intensity. (**c**) Same as Figure 3c, but with VAPB droplets. (**d**) Real-time observation of the transition in VAPB droplet size. The center panel shows the observed *OD*_660_/*OD*_330_ values (open circles) with the best-fit sigmoid curve (black lines) assuming a two-state transition. The transition midpoint time was six hours. The surrounding heatmaps show the real-time estimation of the scattering intensity weighted average of the droplet size during the transition.

Here, we found that the MSP domain of the VAPB protein forms droplets at room temperature under acidic conditions^36^. The droplet size of the VAPB protein was about Φ1,000 nm, as estimated by microscopy (Fig. 4a). The UV‒Vis spectrum of the VAPB droplets in Fig. 4b was flatter, as observed for the larger particles (Figs. 1d and 3b). By the spectral shape fitting (heatmap), the scattering intensity weighted average of the VAPB droplet size was estimated to be around Φ1,500 nm (see Fig. 4c). This value was close to that obtained through microscopic analysis.

### Application of the LLPS size detection method to real-time observation of droplet formation

Previous studies of the droplet formation process have focused primarily on events that occur within one hour and have provided valuable insight into LLPS^37^. However, several hours of measurements remain difficult due to technical limitations such as sample drying and photobleaching. In contrast, our LLPS size detection method was free from sample drying and photobleaching. Thus, it was applicable for up to 12 h of measurements.

Figure 4d (center) shows the time courses of the *OD*_660_/*OD*_330_ values of VAPB droplets, and a clear transition is observed. The transition midpoint time obtained via sigmoidal function fitting was 6.0 h. Figure 4d (surroundings) shows heatmaps that estimate the scattering intensity weighted average of the size of VAPB droplet via spectral shape fitting over time. These results indicate that the scattering intensity weighted average of VAPB droplets size increased monotonically and cooperatively over time. Therefore, our method can capture the slow LLPS formation process in real time for up to 12 h, providing a better understanding of droplet dynamics.

### Droplet fusion

The formation of droplets is often not in a strict equilibrium state, and droplet fusion is frequently observed, as shown in Fig. 4d. Long-term measurements can reveal that droplets sediment over time. An increase in concentration near the bottom of the well increases the droplet fusion rate in that region. In this case, cuvette analysis is difficult to detect regions that change over time because this fusion-related change occurs below the optical path. On the other hand, microplate analysis, in principle, is able to detect whole regions. Therefore, although determining the absolute distributions of droplet size and concentration is difficult, microplate analysis is sensitive for observing droplet size and concentration changes.

### Future challenges

In this experiment, polystyrene plates were used, so measurements were made in the near-ultraviolet and visible light range of 330 nm to 800 nm. The lower limit can be easily extended to approximately 300 nm, where absorption by the aromatic rings of proteins can be effectively ignored. Furthermore, in principle, the method used in this study can be extended to far-ultraviolet and infrared regions, where samples have optical absorption, by taking into account the imaginary term of the refractive index in the calculation of Mie scattering.

If the spectrometer of the microplate reader is configured with a white light source and a diffraction grating and is capable of spectral measurement, this method can be applied as is. On the other hand, if the microplate reader uses filter sets, this method cannot be applied as is, although some particle size information may be obtained by combining two or more filter sets.

As we have seen, under ideal conditions, the scattering intensity weighted average of droplet size can be estimated directly from the spectral shape. However, when there is a particle size distribution, the spectrum generally tends to emphasize the contribution of large particles (Fig. 1e). Furthermore, in practical applications, it is necessary to interpret the spectral shape while considering the non-idealities of the instrument and the effects of multiple scattering (see supplemental text). To this end, it would be useful to obtain spectra for various particle sizes and concentrations of glass beads using each instrument. With our heatmap method, we can estimate the scattering intensity weighted average of the droplet size that most closely matches the spectral shape while accounting for these aspects. In the future, it would be beneficial to consider the effects of instrumental non-ideality and multiple scattering separately and quantitatively. This would allow for a more accurate evaluation of particle size and absolute particle concentration.

## Conclusion

In this study, we developed a simple, quantitative method for estimating the scattering intensity weighted average of the droplet size of LLPS. This method involves analyzing the UV‒Vis scattering/turbidity spectra acquired with the microplate reader. Our approach has several advantages over conventional techniques, such as dynamic light scattering and optical microscopy. It requires only the microplate reader, is compatible with small sample volumes, and allows high-throughput formats. Additionally, it enables real-time monitoring of LLPS dynamics without labeling or specialized optics.

We validated our method using TIA-1 peptide droplets and monitored VAPB protein droplet formation over time. We achieved this by utilizing the dependence of spectral shape on scattering intensity weighted average of the particle size and referencing the UV‒Vis scattering/turbidity spectra of known-size glass beads. Although our method enables us to estimate the relative size of droplets based on spectral shape, it cannot yet provide absolute particle sizes with nanometer-scale precision. The estimated size should be interpreted as the scattering intensity weighted average of the droplet size.

Although some limitations remain, such as the effects of multiple scattering and instrument-specific non-idealities, our findings pave the way for broader applications of spectral, shape-based droplet analysis in studies of LLPS. Future refinement of this approach will enable more accurate particle size quantification and improved compositional and structural heterogeneity discrimination in biomolecular condensates.

## Methods

### Glass and polystyrene beads

The glass beads (silica microsphere beads, white, Sicastar^®^, plane) and the polystyrene beads (polystyrene microsphere beads, white, Micromer^®^, plane/PEG300/COOH) were purchased from Micromod Partikeltechnologie GmbH. Water suspensions of various sizes of glass beads (Φ70, Φ200, Φ400, Φ500, Φ800, Φ1,000, Φ1,500, and Φ3,000 nm) and Φ1,000 nm polystyrene beads were used. For each sample, a stock suspension was prepared with a control concentration of ×1, where the *OD*_330_ value was approximately 1.5. The absolute concentrations of each ×1 sample are as follows, where the unit is particles per mL; Φ3,000 nm: 2.60 × 10^8^, Φ1,500 nm: 7.00 × 10^8^, Φ1,000 nm: 1.71 × 10^9^, Φ800 nm: 2.82 × 10^9^, Φ500 nm: 9.50 × 10^9^, Φ400 nm: 1.88 × 10^10^, Φ200 nm: 3.00 × 10^11^, Φ70 nm: 2.41 × 10^13^. To eliminate variation in particle concentration within the suspensions, the bead solutions were gently stirred by rotating a pipette tip in a circular motion instead of pipetting. For each glass bead suspension, a dilution series was prepared with distilled water to obtain final concentrations of ×0.5, ×0.25, ×0.125, and ×0.0625. To minimize sedimentation and ensure reproducibility, all dilutions were prepared immediately before microplate reader measurements.

### Protein expression and purification

The VAPB gene was cloned and inserted into the pCold-GST vector^38^ and expressed as a GST-fusion protein in *Escherichia coli* BL21 Rosetta (DE3) (Novagen). The cells were cultured in LB medium. The expressed GST-fused protein was purified via glutathione Sepharose 4B resin (GE Healthcare). After removing the GST tag with the HRV 3 C protease, VAPB was further purified by gel filtration chromatography via a Superdex 75 column (GE Healthcare). For more details, see reference^38^.

### TIA-1 peptide sequences

The TIA-1 peptide sequences are as follows. They are designed to have same amino acid composition^30^.

WT: MGSSHHHHHHHHHHHHSENLYFQGGQYVPNGWQVPAYGVYGQPWSQQGF NQTQSSAPWMGPNYSVPPPQGQNGSMLPSQPAGYRVAGYETQ.

L20: MGSSHHHHHHHHHHHHSENLYFQGPMVPGGSASQRNQAQVYYFPYSGPPG SGNQENQTQVWYWPYGAPPGSGNQQSQTQVLYWPVGAPMGQ.

L50: MGSSHHHHHHHHHHHHSENLYFQGQPEGGGGVYYWFLQSSSNPVQAQPQV PPQMNAPQMSYGPTRAQPGYAQPQPNGGGGVYYWWVQSSN.

NP: MGSSHHHHHHHHHHHHSENLYFQGGNMAQLPGQVQMGGQSVPGSPSYQVN QPSYGRYASNWGQPQPAGTYPQQPVGNQFVEPGTYSYPAWW.

### Phase separation experiments

For the TIA-1 peptide phase separation experiments, droplets were formed by diluting a stock dimethyl sulfoxide (DMSO) solution of 3 mM TIA-1 peptide with the following buffer to 25 µM (final concentration). The buffer consisted of 50 mM potassium phosphate (pH 6.8), 50 mM potassium chloride (KCl), and 1 mM dithiothreitol (DTT) (final concentration). To regulate droplet size, this diluted solution was heated at 95 °C for 1 min via a heating block, cooled to room temperature for 20 min, and subjected to microplate measurements.

For the VAPB protein phase separation experiments, the purified VAPB protein was concentrated and buffer exchanged against potassium acetate (CH_3_COOK) buffer (pH 4.7) using Amicon Ultra centrifugal filter units (Millipore) with a 10-kDa molecular mass cutoff in a swinging bucket rotor. Phase-separation reactions were performed on microplates for a final concentration of 0.05 mM VAPB in the 25 mM potassium acetate buffer (pH 4.7, 50 mM KCl, and 0.5 mM DTT) and 15% (w/v) polyethylene glycol (PEG) 8000 (Sigma‒Aldrich). Before the phase separation reactions, each solution of PEG, VAPB protein, and buffer was preincubated at 4 °C for 15 min. For all LLPS reactions, 30% PEG was added dropwise from above without touching the liquid surface, taking care not to shock the sample (final PEG concentration was 15%). The mixtures of all the solutions were subsequently incubated for 24 h at 4 °C to allow for phase separation.

### Microscopy

Microscopic observations were performed with phase-contrast bright-field microscopy using a Leica DMI3000B microscope (Leica) equipped with an N PLAN 20X/0.35 objective lens (Leica) and a phase-contrast condenser PH1 for image analysis. The contrast was enhanced via ImageJ, and the scale bar was generated via Leica LAS-X software.

### Microplate reader measurement

For the spectral intensity measurement of the glass/polystyrene beads, the beads were mixed with distilled water in a 96-well microplate (polystyrene flat bottom, AS ONE) and measured in microplate reader. The UV‒Vis spectral intensities, as optical density (*OD*) values, were measured in the 330–800 nm range with a bandwidth of 5 nm using a Varioskan Flash 2.4 (Thermo Scientific). LLPS was induced as described above for 24 h at 4 °C in a total of 200 µl of solution per well on the microplate. Consequently, the path length for the scattering experiment is 6.84 mm. To monitor the LLPS formation process, the spectral intensity was measured every hour for 12 h, starting immediately after the microplate was prepared by adding drops of PEG solution to the protein mixture.

### Simulation of Lorentz-Mie-Debye scattering

The UV‒Vis scattering/turbidity spectra were simulated for various particles, based on their respective scattering cross-sections at each wavelength. These cross-sections were calculated using the Mie Scattering Version 2-6-3 software developed by Scott Prahl^39^. The refractive indices of water^40^, glass^41^, and polystyrene^42^ were obtained from the literature. The refractive index of the protein solution was calculated under the assumption that the refractive index increment^43^ of the urease solution relative to the refractive index of water is constant over the entire wavelength range and that the particles contain urease at a molecular weight of approximately 100,000 and a concentration of 1 mM. Since absorption is virtually negligible for water, glass, polystyrene, and colorless protein in the range of 330 to 800 nm, the imaginary part of the refractive index was set to zero in this study. A table of refractive indices and calculation methods for bovine serum albumin (BSA) solution and PEG-8000 are given in the supplementary materials.

### Estimation of droplet size using a microplate reader

UV‒Vis spectra (spectral intensity) were recorded as optical density (*OD*) values at 10-nm intervals across the 330–800 nm range. Spectral shape data from eight glass beads of various sizes (Φ70, Φ200, Φ400, Φ500, Φ800, Φ1,000, Φ1,500, and Φ3,000 nm) were used as reference scattering profiles corresponding to known particle sizes. The root mean square difference (RMSD) between the *OD* values of each sample and each reference glass bead spectrum was calculated over the 330–800 nm range to estimate the size of the LLPS droplets. The absolute values of each reference glass bead spectrum were linearly scaled to minimize the RMSD values. The resulting RMSD values were visualized as a heatmap, with glass bead size on the vertical axis and concentration on the horizontal axis. The glass bead size that most closely resembled the scattering pattern of the LLPS sample was identified based on the minimum RMSD values. This size was defined as the scattering intensity weighted average size (diameter) of the LLPS sample.

### Estimation of the transition midpoint time

Since the spectral shape change is potentially monitored by the ratio of spectral intensities at two different wavelengths, the ratio of the *OD* values at 660 and 330 nm (*OD*_660_/*OD*_330_) was employed. 660 nm was selected as the longest wavelength commonly used to monitor turbidity, and 330 nm was selected as the shortest wavelength monitored in our experiment. To evaluate the transition midpoint time, the *OD*_660_/*OD*_330_ values were fitted with the following sigmoid curve:

$$\:{OD}_{660}\:/\:{OD}_{330}\:\left(t\right)=({A}_{1}-{A}_{2})/\left\{1+{(t/{t}_{0})}^{p}\right\}+{A}_{2}$$

where *A*_1_, *A*_2_, *t*_0_, and *p* are the initial and final *OD*_660_/*OD*_330_ values, the transition midpoint time, and the exponent of the transition function, respectively.

## Supplementary Information

Below is the link to the electronic supplementary material.

Supplementary Material 1

Supplementary Material 2

## Data Availability

The datasets and materials used and/or analyzed during the current study available from the corresponding authors on reasonable request.
