# Supplementary material for: LLPS droplet size estimation via UV–Vis spectroscopy using a microplate reader
Source: Sci Rep. 2025 Dec 27;16:3533. doi: 10.1038/s41598-025-33638-8 (PMC12847920; doi:10.1038/s41598-025-33638-8)
Supplement: Supplementary file 1 — Supplementary Material 1 [file 41598_2025_33638_MOESM1_ESM.pdf]

Supporting Information

LLPS droplet size estimation via UV-Vis spectroscopy using a microplate reader

Mayu Enomoto-Kusano[1,#], Takashi S. Kodama[2,#,*], Suai Anzawa[1], Kyoko Furuita[2], Ryoga Kobayashi[3], Naotaka Sekiyama[3], Wataru Togawa[1], Toshimichi Fujiwara[2], Yohei Miyanoiri[2], Hidehito Tochio[3], Chojiro Kojima[1,2,*]

[1]Graduate School of Engineering Science, Yokohama National University, Tokiwadai 79-5, Hodogaya-ku, Yokohama, 240-8501, Japan,
[2]Institute for Protein Research, The University of Osaka, 3-2 Yamadaoka, Suita, Osaka, 565-0871, Japan,
[3]Department of Biophysics, Graduate School of Science, Kyoto University, Kitashirakawa Oiwake-cho, Sakyo-ku, Kyoto, 606-8502, Japan.

*To whom correspondence should be addressed. Chojiro Kojima (E-mail, kojima-chojiro-xk@ynu.ac.jp; Tel, +81-45-339-4232) and Takashi S. Kodama (E-mail, tskodama@protein.osaka-u.ac.jp; Tel, +81-6-6879-4417).

Supplementary Information Contents

Supplementary Text
Supplementary references
Tables S1-S8
Figures S1–S8
Refractive Indices Table.xlsx (attached file)

Supplementary Text

This study proposes a method for evaluating the particle size of suspended particles in a solution by measuring turbidity at multiple wavelengths. Here, we briefly explain the background of the theoretical formulas used in the main text. For details, see the textbooks[1-4].

1. Turbidity and scattering cross section

As shown in the main text, the optical density *OD* at the wavelength λ_0, an actual observable, is determined by turbidity τ and the optical path length l. Turbidity τ depends on the density (concentration) of the suspended particles N, and the scattering cross section C_{sca}. Here, λ_0 is the wavelength of light in a vacuum and is expressed to distinguish it from the wavelength λ in a medium.

$$OD(\lambda_0) = -\,log\left(\frac{I}{I_0}\right) = \frac{\tau l}{ln10} = \frac{NC_{ext}l}{ln10} \ . \qquad \text{[Eq. S1]}$$

According to the law of conservation of energy, the extinction of light is the sum of the contributions of absorption and scattering. Therefore this extinction cross section C_{ext} is expressed as the sum of the scattering cross section C_{sca} and the absorption cross section C_{abs}:

$$C_{ext} = C_{sca} + C_{abs} \ . \qquad \text{[Eq. S2]}$$

Therefore, in the wavelength range where neither the suspended particles nor the surrounding medium absorb light, the extinction cross section is equal to the scattering cross section:

$$C_{ext} = C_{sca} \ , \qquad \text{[Eq. S3]}$$

where the scattering cross section C_{sca} is a complex function of the size and shape of the scatterer, the complex refractive indices of the scatterer and the surrounding medium, and the incident light wavelength.

Furthermore, the scattering cross section at each wavelength differs for each particle size in a mixture of particles of multiple sizes. However, these cross sections contribute independently to the overall turbidity under conditions where multiple scattering can be ignored; thus, simple additivity holds.

$$OD(\lambda_0) = \frac{Nl}{ln10}\sum C_{sca,i}f_i \ , \qquad \text{[Eq. S4]}$$

where $C_{sca,i}$ is the scattering cross section of the particle with the i-th size, f_i is the fraction of particles of that size among the total, and N is the total number

of scattering particles.

2. Derivation of the scattering cross section

We briefly explain the theoretical background of how the scattering cross section C_{sca} is derived, assuming that the scatterers are spherical particles with a uniform refractive index and that light is not absorbed by the particles and the surrounding medium. Furthermore, the spatial spacing between the scatterers is assumed to be sufficiently large compared to the size of the scatterer itself, and the scattering is a single scattering.

The wave equation for the scattering of a plane wave by a homogeneous spherical particle is originally written down from Maxwell's equations. Equations expressing the angular dependence of the scattering intensity and polarization state can be derived by seeking solutions that satisfy the boundary conditions for continuity between the interior and exterior at the particle surface.

Ultimately, we obtain the relationship between the scattering cross section C_{sca}, which is essential for describing the quantities observed in turbidity measurements, and the particle size, refractive indices, and wavelength. Here, we mainly describe the part after the solution to the wave equation is obtained.

2-1 Scattering angle and plane

Scattering angle θ is the angle from the direction of the incident light propagation to the direction of the observed scattered light. Simultaneously, it defines the scattering plane or observation plane, which is defined by the incident light's propagation direction and the scattered wave's propagation direction.

2-2 Intensity function and scattering coefficient

In order to obtain the value of the scattering cross section C_{sca}, which is the objective here, it is necessary to quantitatively show how much the intensity of the light in the direction of propagation of the incident light is attenuated compared to when there are no suspended particles, for light in the far field, which is sufficiently far from the scatterer.

The intensity of the scattered light towards the scattering angle θ in the far field at a distance R from the scatterer can be expressed as follows using the formula for the components of the incident intensity:

$$I_1^{sca}(\theta) = I_1^{inc} \frac{i_2}{k^2R^2} \ , \qquad \text{[Eq. S5]}$$

$$I_2^{sca}(\theta) = I_2^{inc} \frac{i_1}{k^2R^2} \ , \qquad \text{[Eq. S6]}$$

where the subscripts 1 and 2 represent the parallel and perpendicular components of the electric field oscillation direction relative to the scattering plane, and the superscripts inc and sca distinguish between the intensity of the incident and scattered light, respectively, and k is the propagation constant defined as $k = 2\pi/\lambda$.

The intensity functions i_1 and i_2 are functions of the scattering angle θ and are defined using the amplitude functions $S_1(\theta)$ and $S_2(\theta)$ as follows:

$$i_1 = |S_1(\theta)|^2 \ , \qquad \text{[Eq. S7]}$$

$$i_2 = |S_2(\theta)|^2 \ . \qquad \text{[Eq. S8]}$$

Since these parallel and perpendicular components of scattered light are thought to arise from components of the incident light polarized in the same direction as each component, when the incident light is natural (unpolarized) light, the observable quantity for forward scattering in the far field at θ=0 is simply the arithmetic mean of both components due to symmetry.

If the incident light is natural light ($I_1^{inc} = I_2^{inc} = I_0$) and the scatterer is spherical,

$$I(\theta) = \frac{1}{2}\left(I_1^{inc} \frac{i_2}{k^2R^2} + I_2^{inc} \frac{i_1}{k^2R^2}\right) = \frac{1}{2}\left(\frac{i_2+i_1}{k^2R^2}\right) I_0 \ . \qquad \text{[Eq. S9]}$$

In particular, at $\theta = 0$,

$$S_1(0) = S_2(0) = S(0) \ . \qquad \text{[Eq. S10]}$$

The amplitude functions $S_1(\theta)$ and $S_2(\theta)$ are expressed as follows:

$$S_1(\theta) = \sum_{n=1}^{\infty} \frac{2n+1}{n(n+1)} [a_n \pi_n(\cos\theta) + b_n \tau_n(\cos\theta)] \ , \qquad \text{[Eq. S11]}$$

$$S_2(\theta) = \sum_{n=1}^{\infty} \frac{2n+1}{n(n+1)} [a_n \tau_n(\cos\theta) + b_n \pi_n(\cos\theta)] \ , \qquad \text{[Eq. S12]}$$

where π_n and τ_n are the angular functions given by the following equations using the associated Legendre polynomial $P_n^{(1)}$ and its derivative with respect to the scattering angle θ.

$$\pi_n(\cos\theta) = \frac{1}{\sin\theta} P_n^{(1)}(\cos\theta) \ , \qquad \text{[Eq. S13]}$$

$$\tau_n(\cos\theta) = \frac{d}{d\theta} P_n^{(1)}(\cos\theta) \ . \qquad \text{[Eq. S14]}$$

Furthermore, a_n and b_n are called scattering coefficients and are defined as follows:

$$a_n = \frac{\psi_n'(\beta)\psi_n(\alpha) - m\psi_n(\beta)\psi_n'(\alpha)}{\psi_n'(\beta)\zeta_n(\alpha) - m\psi_n(\beta)\zeta_n'(\alpha)} \ , \qquad \text{[Eq. S15]}$$

$$b_n = \frac{m\psi_n'(\beta)\psi_n(\alpha) - \psi_n(\beta)\psi_n'(\alpha)}{m\psi_n'(\beta)\zeta_n(\alpha) - \psi_n(\beta)\zeta_n'(\alpha)} \ , \qquad \text{[Eq. S16]}$$

where ψ_n and ζ_n are Ricatti-Bessel functions, and ψ_n' and ζ_n' represent their derivatives, respectively. The arguments α and β are the size parameter and the size parameter multiplied by the relative refractive index, respectively, and are defined as follows using the particle radius r, the particle refractive index m_1, the refractive index of the surrounding medium m_2, the relative refractive index defined m, the light propagation constant k_1 within the particle, and the propagation constant k_2 within the medium, respectively:

$$\alpha = k_2 r \ , \qquad \text{[Eq. S17]}$$

$$\beta = m\alpha \ , \qquad \text{[Eq. S18]}$$

$$k_1 = \frac{2\pi m_1}{\lambda_0} \ , \qquad \text{[Eq. S19]}$$

$$k_2 = \frac{2\pi}{\lambda} = \frac{2\pi m_2}{\lambda_0} \ , \qquad \text{[Eq. S20]}$$

$$m = \frac{m_1}{m_2} = \frac{k_1}{k_2} \ . \qquad \text{[Eq. S21]}$$

When describing the scattering theory in this manner, we use the size parameter is used instead of the particle radius or diameter, which is normalized by wavelength. Also, if there is absorption inside the particle, then m_1 is a complex number, and so is m. However, if there is no absorption, both are real numbers.

Specifically, Ricatti-Bessel functions can be expressed using Bessel functions (Bessel functions of the first kind), Neumann functions (Bessel functions of the second kind), and Hankel functions (Bessel functions of the third kind), as shown below.

$$\psi_n(z) = z j_n(z) = \left(\frac{\pi z}{2}\right)^{1/2} J_{n+\frac{1}{2}}(z) \ , \qquad \text{[Eq. S22]}$$

$$\chi_n(z) = -z n_n(z) = \left(\frac{\pi z}{2}\right)^{1/2} N_{n+\frac{1}{2}}(z) \ , \qquad \text{[Eq. S23]}$$

$$\zeta_n(z) = zh_n^{(2)}(z) = \left(\frac{\pi z}{2}\right)^{1/2} H_{n+\frac{1}{2}}^{(2)}(z) = \psi_n(z) + i\chi_n(z) \ , \quad \text{[Eq. S24]}$$

$$\zeta_n^{(1)}(z) = zh_n^{(1)}(z) = \left(\frac{\pi z}{2}\right)^{1/2} H_{n+\frac{1}{2}}^{(1)}(z) = \psi_n(z) - i\chi_n(z) \ , \text{[Eq. S25]}$$

where $J_{n+\frac{1}{2}}(z)$, $N_{n+\frac{1}{2}}(z)$, and $H_{n+\frac{1}{2}}(z)$ are the half-integral order Bessel, Neumann and Hankel functions, respectively. $j_n(z)$, $n_n(z)$, and $h_n(z)$ are the corresponding spherical Bessel (Spherical Bessel functions of the first kind), Neumann (Spherical Bessel functions of the second kind), and Hankel (Spherical Bessel functions of the third kind) functions, respectively. The Hankel functions of the first or second kind are distinguished by superscripts.

2-3 Scattering coefficient and scattering cross section

If the particles absorb or scatter light, the attenuation of light passing through the suspension is described by a value called the extinction cross section:

$$C_{ext} = \left(\frac{\lambda^2}{2\pi}\right) \sum_{n=1}^{\infty} (2n+1)\{Re(a_n + b_n)\} \ . \quad \text{[Eq. S26]}$$

However, in the wavelength region where neither the suspended particles nor the surrounding medium absorb light, this extinction cross section coincides with the scattering cross section, so the extinction cross section C_{ext} is the same as the scattering cross section C_{sca}:

$$C_{sca} = \left(\frac{\lambda^2}{2\pi}\right) \sum_{n=1}^{\infty} (2n+1)\{|a_n|^2 + |b_n|^2\} \ . \quad \text{[Eq. S27]}$$

In order to calculate the scattering cross section at a specific wavelength when particles with a specific particle size and refractive index are dispersed in a specific medium, it is necessary to numerically calculate the Ricatti-Bessel functions ψ_n, ζ_n and their derivatives ψ_n', ζ_n' to obtain the scattering coefficients a_n and b_n, and the associated Legendre polynomial $P_n^{(1)}$ and it's derivative in order to obtain the angular functions π_n and τ_n.

In recent years, many efficient algorithms and programs have become available for these numerical calculations[5]. Historically, the most widely used program is probably the MIEV0 written by Wiscombe[6], which provides accurate results, from Rayleigh limits to very large particles with the size parameter of α up to 10,000/20,000[5,7]. This parameter is defined by [Eq. S17] and [Eq. S20]. At a wavelength of 300 nm, α corresponds to approximately 10 times the particle size;

at a wavelength of 800 nm, α corresponds to approximately four times the particle size.

3-1 Limitation of the LLPS size detection method – Multiple scattering

The theoretical treatment in this study is based on the premise that the particle density in the suspension is low enough to consider scattering as independent single scattering. However, as the particle density increases, the effect of multiple scattering, in which scattered light is further scattered in the light path, gradually becomes impossible to ignore (Figure S7). The effect of multiple scattering appears as the particle concentration increases, and since it is larger the stronger the forward scattering, it becomes more noticeable when the particle size is large, or the wavelength is short. Empirically, in UV-Vis spectral region, if the OD value is less than 0.04[1] (significantly less than 0.43[3]), it can be considered as single scattering. If the OD value is between 0.04 and 0.13, double scattering correction may be necessary. If the OD value is greater than 0.13, multiple scattering must be considered. In the case of the experimental value of glass particles, the condition of single scattering is met when the sample concentration is low and the measurement is in the long wavelength region, or when the particle size is small. However, when the particle size is large or the concentration is high, it is difficult to eliminate the effect of multiple scattering completely.

3-2 Limitation of the LLPS size detection method – Instrument non-ideality

Another issue is the effect of non-ideality of instruments. Plate readers are devices designed to measure absorption and fluorescence spectra and are not specifically designed to evaluate extinction due to light scattering. Generally, if the observation system intercepts a portion of the forward-scattered light at a low scattering angle, it misinterprets it as transmitted light intensity[2,8]. Therefore, there is a risk that the instrument constants may affect the spectral shape because the detector systematically detects this forward scattered light at a certain solid angle.

The extent to which this non-ideality affects the measurement depends on the relationship between the phase function of the scattered light (i.e., the angular distribution of scattered light intensity), the OD values, and the arrangement of the optical elements. As shown in Figure S8, larger particle sizes

result in a higher percentage of forward scattering, as indicated by the shape of the phase function. Additionally, the shorter the wavelength, the more concentrated the scattered light is in the forward direction. The degree of the effect varies with the absolute value of the transmitted light intensity (i.e., the OD value). Thus, the effect of the non-ideality of the observation system depends on the particle size and its absolute concentration.

As shown in the main text, the spectral shapes of glass bead suspensions measured at different concentrations did not completely overlap even after normalizing the intensities. This is thought to reflect the multiple scattering problems mentioned above and the problem of the non-ideality of the detection angle of the detector.

4-1 Refractive Index of Bovin Serum Albumin (BSA) Solution

Proteins' refractive index is affected by their amino acid composition[9] and conformation[10]. Therefore, accurately measuring the refractive index of various proteins is difficult, and few reports on chromatic dispersion. Since it is generally difficult to measure the refractive index n(λ) at multiple wavelengths, a theoretical approach using the Kramers-Kronig (K-K) relation[11] is used as an alternative. In this study, the refractive index of bovine serum albumin (BSA) aqueous solution in the range of 300–800 nm was estimated based on the imaginary term of the complex refractive index of BSA measured in the vacuum ultraviolet region[12].

The refractive index of the BSA sample obtained by this calculation corresponds to 100% BSA in mass fraction, so n(λ) in an aqueous solution of any concentration was further calculated using the following formula:

$$n(\lambda) = n_{water}(\lambda) + \left(w_{BSA} \times n_{BSA}(\lambda) - n_{water}(\lambda)\right) \text{ , } \qquad \text{[Eq. S28]}$$

where, and represent the refractive index of water, the mass fraction of BSA in solution, and the refractive index of a 100% BSA equivalent sample, respectively, for the wavelength.

In the field of antibody therapy, the volume of subcutaneous antibody injection must be within 1.5 mL, the protein concentration must be 100 mg/mL or higher[13]. It has been reported that concentrations of 170 mg/mL can be achieved in LLPS[14]. Furthermore, the total amount of intracellular molecules is considered to be 400 mg/mL on average[15]. Thus, 100 and 400 mg/mL BSA solutions were selected as representative protein concentrations.

Even peptides with the same amino acid composition have different droplet formation properties, which in principle could lead to differences in the nature of

the intermolecular interactions within the particles, which in principle could affect the refractive index. However, previous study[16] has shown that, although the nature of the interactions varies with polymer concentration, the refractive index varies approximately linearly with concentration. Therefore, such interactions were assumed not to significantly affect the refractive index, and additivity can be assumed.

4-2 Refractive Index of PEG-8000 Solution

The refractive index of a PEG-8000 aqueous solution at the sodium D1 line (589.5224 nm) was calculated using the following method[16], excluding the effects of coexisting salts.

$$n_{PEG-8000}(\mathrm{D1}) = n_{water}(\mathrm{D1}) + a_{PEG-8000} \times w_{PEG-8000} \ , \qquad \text{[Eq. S29]}$$

where $n_{water}(\mathrm{D1})$ is D1 line refractive index of water at 298.2 K (1.3325), $a_{PEG-8000}$ is the concentration coefficient of PEG-8000 (0.1328), and $w_{PEG-8000}$ is the PEG-8000 mass fraction. From this calculation, the refractive index of a 100% PEG-8000 equivalent sample at the sodium D1 line, $n_{PEG-8000}(\mathrm{D1})$, is 1.4653 at 298.2 K. This corresponds to 1.34252 for a 15% PEG-8000 aqueous solution.

Since the literature values for the wavelength dispersion of the refractive indecies of PEG-8000 solution were unknown, we calculated them using the refractive index of PEG-8000 solution at the D1 line described above and the literature values for the wavelength dispersion of the refractive indecies of PEG-400. Specifically, in the wavelength range from 300 to 800 nm, the refractive indecies of PEG-8000 solution, $n_{P8000}(\lambda)$, was calculated using the following equation:

$$n_{PEG-8000}(\lambda) = \left(n_{PEG-400}(\lambda) - n_{water}(\lambda)\right) \times \left(w_{PEG-8000} \times n_{PEG-8000}(D1) - n_{PEG-400}(D1)\right) \ , \qquad \text{[Eq. S30]}$$

where $n_{PEG-8000}(D1)$ is the value calculated above (1.34252), $n_{PEG-400}(D1)$ is the literature value for the refractive index of PEG-400 at the D1 line[17] (1.4649), $n_{water}(\lambda)$ is the refractive index of water[18] at wavelength λ, and $n_{PEG-400}(\lambda)$ is the refractive index of PEG-400 at wavelength λ. We assume that $n_{PEG-400}(\lambda)$ can be calculated using the following formula, and each coefficient was obtained by fitting the refractive indecies of PEG-400 at multiple wavelengths to literature values[17].

$$n_{PEG-400}(\lambda) = a\lambda^2 + b\lambda + c \ . \qquad \text{[Eq. S31]}$$

The optimized coefficients are: a=7.330910339165E-08,
b=-0.000131709741203259, and c=1.51737463758106.

4-3 Refractive Index of Bovin Serum Albumin (BSA) with PEG-8000 Solution

The refractive index of the mixed solution of BSA and PEG at wavelength λ was calculated assuming the following relationship:

$$n(\lambda) = n_{water}(\lambda) + \left(w_{PEG-8000} \times n_{PEG-8000}(\lambda) - n_{water}(\lambda)\right) + \left(w_{BSA} \times n_{BSA}(\lambda) - n_{water}(\lambda)\right) \quad ,$$

[Eq. S32]

where $n_{water}(\lambda)$, $w_{PEG-8000}$, $n_{PEG-8000}(D1)$, w_{BSA}, and $n_{BSA}(\lambda)$ represent the refractive index of water, the mass fraction of PEG-8000, the refractive index of a 100% PEG-8000 equivalent sample, the mass fraction of BSA, the refractive index of a 100% BSA equivalent sample, respectively for the wavelength λ, as described above.

Here too, we assume linear additivity for the contribution of each component to the refractive index. If there is interaction between BSA, PEG, and water, perturbations will occur in the molecular orbitals of their respective outermost electrons, which in principle could affect the refractive index. However, for the same reasons as in the discussion of the concentration-dependent change in the refractive index of BSA above, we believe that such interactions do not have a significant effect on the refractive index, and therefore it is reasonable to assume additivity.

Supplementary references

1. van de Hulst, H. C. *Light scattering by small particles* (John Wiley and Sons, 1957).
2. Kerker, M. *The scattering of light and other electromagnetic radiation* (Academic Press, 1969).
3. Bohren, C. F. & Huffman, D. R. *Absorption and scattering of light by small particles* (Wiley-VCH, 1983).
4. Liou, K. N. *An introduction to atmospheric radiation* (Academic Press, 2002).
5. Wriedt, T. Mie theory: a review in *The Mie theory* (ed. Hergert, W. & Wriedt, T.) 53–71 (Springer, 2012).
6. Wiscombe, W. J. Mie scattering calculations: advances in technique and fast, vector-speed computer codes. *NCAR/TN-140+STR, NCAR Tech. Note* (National Center for Atmospheric Research, 1979).
7. Hong Du, Mie-scattering calculation, *Appl. Opt.* **43**, 1951-1956 (2004)
8. Hinds, W. C. *Aerosol technology: properties, behavior, and measurement of airborne particles* (John Wiley and Sons, 1982).
9. Zhao, H., Brown, P. H. & Schuck, P. On the distribution of protein refractive index increments. *Biophys. J.* **100**, 2309-2317 (2011).
10. Khago, D., Bierma, J. C., Roskamp, K. W., Kozlyuk, N. & Martin, R. W. Protein refractive index increment is determined by conformation as well as composition *J. Phys.: Condens. Matter* **30**, 435101 (2018).
11. Benatto, L. et al. RI−Calc: A user friendly software and web server for refractive index calculation. *Comput. Phys. Commun.* **298**, 109100 (2024).
12. Inagaki, T., Hamm, R. N., Arakawa, E. T. & Birkhoff, R. D. Optical property of bovine plasma albumin between 2 and 82 eV. *Biopolymers* **14**, 839-847 (1975).
13. Shire, S. J., Shahrokh, Z. & Liu, J. Challenges in the development of high protein concentration formulations. *J. Pharm. Sci.* **93**, 1390-1402 (2004).
14. Bramham, J. E., Davies, S. A., Podmore, A. & Golovanov, A. P. Stability of a high-concentration monoclonal antibody solution produced by liquid–liquid phase separation. *mAbs* **13**, 1940666 (2021).
15. Bai, Q., Liu, Z., Chen, J. & Liang, D. Crowded environment regulates the coacervation of biopolymers via nonspecific interactions. *Biomacromolecules* **24**, 283-293 (2023).
16. Huang, Q., Li, L., Li, M., Wang, L. & Yu, X. Measurements and thermodynamic modeling of solid–liquid equilibria data for the ternary

(KCl + PEG8000 + H2O) system at 288.2, 298.2, and 308.2 K. *J. Solution Chem.* **50**, 792–807 (2021).

17. Tuchina, D. K., Genin, V. D., Bashkatov, A. N., Genina, E. A. & Tuchin, V. V. Optical clearing of skin tissue ex vivo with polyethylene glycol. *Opt. Spectrosc.* **120**, 28–37 (2016).
18. Segelstein, D. J. The complex refractive index of water. *Master's Thesis* (University of Missouri-Kansas City, 1981).

Table S1. UV-Vis spectral intensity data for Φ3,000 nm glass beads.

**Φ3000 nm**	Spectral Intensity [OD]				
Wavelength [nm]	×1	×0.5	×0.25	×0.125	×0.0625
330	1.426598	1.031122	0.693975	0.404247	0.224422
340	1.423248	1.028438	0.692266	0.403431	0.22433
350	1.419352	1.02552	0.690159	0.402337	0.223898
360	1.414446	1.022519	0.688077	0.4011	0.223375
370	1.411752	1.019837	0.686018	0.400316	0.223428
380	1.406512	1.015136	0.682405	0.397692	0.221955
390	1.400489	1.009999	0.678075	0.394501	0.21989
400	1.395107	1.004937	0.673893	0.391594	0.217953
410	1.389718	1.000329	0.669888	0.388324	0.215851
420	1.384088	0.995563	0.665499	0.384813	0.213427
430	1.379899	0.992051	0.662104	0.382142	0.211714
440	1.374823	0.987164	0.65851	0.379622	0.210232
450	1.370212	0.983002	0.654582	0.376757	0.208411
460	1.364873	0.978688	0.650693	0.373785	0.206496
470	1.360689	0.975337	0.647828	0.371726	0.205193
480	1.356285	0.971424	0.644301	0.369029	0.203472
490	1.352354	0.967904	0.64103	0.366312	0.202039
500	1.347745	0.964103	0.637697	0.363991	0.20046
510	1.343008	0.960043	0.634246	0.361463	0.198653
520	1.338842	0.956441	0.630964	0.358966	0.197483
530	1.33458	0.952213	0.627662	0.356524	0.195938
540	1.330863	0.948804	0.624157	0.354371	0.194808
550	1.326781	0.94549	0.621291	0.352321	0.19341
560	1.323018	0.941742	0.618415	0.350439	0.192349
570	1.318752	0.93858	0.615277	0.347764	0.191263
580	1.315131	0.935099	0.612477	0.345833	0.189985
590	1.311159	0.931768	0.60949	0.343739	0.188895
600	1.307386	0.928561	0.606346	0.341611	0.187677
610	1.303668	0.925113	0.603056	0.339071	0.186427
620	1.299581	0.921461	0.600154	0.337478	0.185423
630	1.295728	0.918376	0.597342	0.335345	0.184379
640	1.292136	0.915245	0.594409	0.333428	0.183428
650	1.288543	0.911763	0.591755	0.331624	0.182532
660	1.284738	0.908491	0.58856	0.329392	0.181375
670	1.281566	0.905355	0.585543	0.327722	0.180577
680	1.277404	0.902028	0.582863	0.325626	0.179575
690	1.274355	0.899367	0.580292	0.323503	0.178401
700	1.270759	0.895748	0.577273	0.32162	0.177388
710	1.266639	0.892917	0.575586	0.321852	0.177553
720	1.264785	0.889692	0.570606	0.315688	0.174787
730	1.261117	0.886292	0.567716	0.314619	0.174088
740	1.257453	0.883762	0.566398	0.315116	0.174337
750	1.254081	0.880611	0.563692	0.313268	0.173323
760	1.250577	0.877582	0.5605	0.311084	0.172512
770	1.247826	0.874501	0.557544	0.309014	0.171535
780	1.244015	0.870634	0.554347	0.306571	0.169986
790	1.240583	0.867119	0.551229	0.304911	0.169155
800	1.23776	0.864586	0.549041	0.302954	0.16863

Table S2. UV-Vis spectral intensity data for Φ1,500 nm glass beads.

**Φ1500 nm**	Spectral Intensity [OD]				
Wavelength [nm]	×1	×0.5	×0.25	×0.125	×0.0625
330	1.462319	1.098823	0.707776	0.44745	0.236378
340	1.459362	1.096009	0.705833	0.446103	0.236563
350	1.455381	1.091799	0.702612	0.442793	0.236023
360	1.452145	1.090193	0.701663	0.444043	0.236371
370	1.448372	1.085156	0.697598	0.439217	0.235862
380	1.442774	1.079472	0.692874	0.434474	0.233638
390	1.436605	1.073099	0.68751	0.43044	0.231701
400	1.431105	1.067583	0.682702	0.427293	0.230223
410	1.425142	1.061412	0.677372	0.423042	0.228473
420	1.419394	1.055809	0.672591	0.420354	0.226483
430	1.414314	1.049438	0.666922	0.414589	0.22463
440	1.409168	1.044481	0.662479	0.412643	0.223556
450	1.403543	1.038793	0.657634	0.40903	0.221823
460	1.398244	1.032746	0.652737	0.405983	0.220266
470	1.392992	1.027103	0.647761	0.402402	0.218869
480	1.388092	1.022012	0.643299	0.399551	0.217376
490	1.383071	1.015973	0.637833	0.395055	0.215836
500	1.378097	1.010583	0.633474	0.392035	0.214755
510	1.372877	1.005054	0.628708	0.389762	0.213203
520	1.368302	0.99964	0.624182	0.386995	0.211802
530	1.363309	0.994258	0.619621	0.383703	0.210339
540	1.35882	0.988802	0.614983	0.380692	0.208638
550	1.353661	0.982815	0.609682	0.376254	0.206884
560	1.348389	0.977515	0.605226	0.37372	0.205557
570	1.343471	0.97179	0.600327	0.370383	0.203781
580	1.338466	0.965702	0.595562	0.366175	0.201862
590	1.333783	0.960432	0.590951	0.363877	0.200181
600	1.329017	0.9546	0.586	0.359602	0.198358
610	1.324476	0.949276	0.581174	0.3566	0.196491
620	1.319577	0.943674	0.576556	0.35339	0.19462
630	1.314245	0.937738	0.571788	0.349722	0.192924
640	1.309049	0.931638	0.567213	0.345883	0.190765
650	1.304006	0.926237	0.56227	0.342618	0.189027
660	1.298718	0.920134	0.557544	0.338835	0.186995
670	1.293986	0.913694	0.552222	0.334497	0.184858
680	1.289013	0.908216	0.547516	0.331271	0.182802
690	1.284271	0.901885	0.542174	0.326971	0.180452
700	1.27961	0.89611	0.537245	0.323537	0.178599
710	1.273209	0.890796	0.532823	0.321001	0.176082
720	1.270124	0.882071	0.527014	0.315373	0.174334
730	1.264437	0.875811	0.521881	0.311814	0.17205
740	1.258141	0.869968	0.516557	0.307333	0.169802
750	1.25254	0.863479	0.511741	0.303885	0.167667
760	1.247428	0.856749	0.506419	0.300419	0.165744
770	1.241974	0.849795	0.50119	0.295595	0.163727
780	1.237013	0.843053	0.496066	0.292441	0.161377
790	1.231399	0.835913	0.490627	0.289022	0.159101
800	1.226305	0.829638	0.485971	0.285368	0.157265

Table S3. UV-Vis spectral data for Φ1,000 nm glass beads.

**Φ1000 nm**	Spectral Intensity [OD]				
Wavelength [nm]	×1	×0.5	×0.25	×0.125	×0.0625
330	1.392996	0.908668	0.548619	0.348238	0.194378
340	1.388772	0.903095	0.543943	0.345699	0.193989
350	1.384706	0.897032	0.538868	0.34229	0.191223
360	1.379115	0.892267	0.535024	0.341121	0.193685
370	1.373975	0.885101	0.529367	0.336572	0.188765
380	1.367583	0.876476	0.521905	0.331882	0.185415
390	1.36018	0.868059	0.514869	0.32704	0.182632
400	1.352167	0.859511	0.508088	0.322348	0.180497
410	1.344176	0.850946	0.501265	0.317738	0.177263
420	1.335872	0.842813	0.494716	0.313753	0.176118
430	1.329899	0.835202	0.48897	0.308516	0.170927
440	1.322334	0.826909	0.482154	0.304673	0.169768
450	1.315338	0.819744	0.476387	0.300253	0.16662
460	1.307473	0.811694	0.470117	0.29624	0.164608
470	1.300241	0.80409	0.464543	0.291617	0.160886
480	1.292509	0.795963	0.458259	0.287546	0.159056
490	1.28591	0.788002	0.451834	0.282276	0.154709
500	1.27911	0.780519	0.445996	0.278223	0.152266
510	1.271503	0.772474	0.439738	0.273862	0.150222
520	1.264424	0.764495	0.43365	0.269552	0.147709
530	1.256559	0.756095	0.427274	0.26484	0.144911
540	1.248758	0.747467	0.420676	0.260507	0.14248
550	1.241389	0.739099	0.414614	0.255585	0.138595
560	1.233446	0.730432	0.408232	0.251475	0.136656
570	1.225636	0.722102	0.402	0.247337	0.134088
580	1.218719	0.713658	0.395752	0.242559	0.130703
590	1.210536	0.705043	0.389594	0.238517	0.129017
600	1.202969	0.696294	0.38342	0.233915	0.126008
610	1.195284	0.687671	0.37728	0.229688	0.123565
620	1.186785	0.679264	0.371369	0.225684	0.121365
630	1.178393	0.670144	0.365446	0.221337	0.118692
640	1.169939	0.660948	0.359281	0.217292	0.116185
650	1.160643	0.65212	0.353251	0.213343	0.113952
660	1.151469	0.642629	0.347325	0.20911	0.111483
670	1.141829	0.633318	0.341164	0.204986	0.10893
680	1.132574	0.624143	0.335377	0.201541	0.106898
690	1.123194	0.615184	0.329901	0.197604	0.104099
700	1.113415	0.606234	0.323604	0.193714	0.102613
710	1.103536	0.599195	0.320388	0.190681	0.100811
720	1.093859	0.587162	0.311705	0.186111	0.097558
730	1.083684	0.578331	0.306364	0.182575	0.095615
740	1.073866	0.570504	0.302278	0.179153	0.093123
750	1.063624	0.561573	0.297144	0.175791	0.091625
760	1.05243	0.552657	0.2917	0.172641	0.089868
770	1.042021	0.5431	0.285743	0.168896	0.087614
780	1.030385	0.534612	0.280574	0.165789	0.086324
790	1.0183	0.525937	0.27545	0.163056	0.084967
800	1.007176	0.517162	0.271	0.159977	0.083024

Table S4. UV-Vis spectral intensity data for Φ800 nm glass beads.

**Φ800 nm**	Spectral Intensity [OD]				
Wavelength [nm]	×1	×0.5	×0.25	×0.125	×0.0625
330	1.485916	1.089189	0.686125	0.4044	0.217262
340	1.479625	1.081849	0.678557	0.399289	0.214863
350	1.471131	1.073022	0.669889	0.393412	0.21186
360	1.463525	1.065007	0.661776	0.388175	0.208827
370	1.454728	1.055649	0.652785	0.381681	0.205417
380	1.444974	1.045452	0.642437	0.374325	0.200793
390	1.435112	1.034176	0.631602	0.366601	0.196173
400	1.425332	1.02356	0.621189	0.359276	0.191917
410	1.414779	1.011952	0.610198	0.351572	0.187299
420	1.404278	1.000025	0.59916	0.343679	0.182705
430	1.394104	0.988478	0.588199	0.335884	0.178555
440	1.383875	0.976934	0.577207	0.328855	0.174206
450	1.37417	0.965226	0.566441	0.321327	0.169529
460	1.364434	0.953263	0.555518	0.313844	0.165126
470	1.354882	0.941267	0.544755	0.306628	0.16119
480	1.344292	0.929053	0.533909	0.299825	0.157143
490	1.333395	0.915965	0.522835	0.29266	0.153252
500	1.322184	0.902984	0.512155	0.285439	0.149456
510	1.31061	0.88944	0.501139	0.27877	0.145479
520	1.299109	0.876024	0.490602	0.272373	0.141924
530	1.287382	0.862518	0.479951	0.265458	0.13827
540	1.27609	0.848855	0.46964	0.259357	0.134659
550	1.264107	0.83524	0.459306	0.253181	0.131275
560	1.252415	0.82185	0.449407	0.247284	0.128009
570	1.240354	0.807927	0.439513	0.241494	0.124857
580	1.22765	0.794122	0.429824	0.235654	0.121726
590	1.214303	0.780137	0.420308	0.230193	0.118714
600	1.200144	0.76593	0.410722	0.224628	0.115845
610	1.185776	0.751932	0.401337	0.219326	0.11287
620	1.17062	0.737472	0.392076	0.213835	0.109992
630	1.155052	0.723252	0.383092	0.208789	0.107271
640	1.139189	0.708835	0.37442	0.203795	0.104646
650	1.123596	0.695181	0.365826	0.198946	0.102217
660	1.107685	0.681161	0.357555	0.194285	0.099427
670	1.091576	0.667413	0.349096	0.189577	0.097117
680	1.07566	0.654392	0.34154	0.185209	0.094639
690	1.059981	0.641014	0.333593	0.181058	0.092413
700	1.04379	0.628286	0.325941	0.176637	0.090169
710	1.027566	0.615607	0.318845	0.172667	0.087964
720	1.011664	0.602521	0.312029	0.168522	0.086109
730	0.995487	0.590187	0.30493	0.164708	0.083978
740	0.978314	0.578217	0.298177	0.160815	0.081789
750	0.962077	0.566819	0.291861	0.1574	0.079975
760	0.945588	0.555217	0.285333	0.153938	0.078205
770	0.928888	0.543908	0.279089	0.150307	0.076441
780	0.912778	0.532331	0.272847	0.146797	0.074495
790	0.896116	0.521144	0.266764	0.14379	0.07282
800	0.880598	0.511114	0.261849	0.14103	0.071417

Table S5. UV-Vis spectral intensity data for Φ500 nm glass beads.

Φ500 nm	Spectral Intensity [OD]				
Wavelength [nm]	×1	×0.5	×0.25	×0.125	×0.0625
330	1.432122	0.915616	0.512932	0.260202	0.139406
340	1.419539	0.895032	0.496511	0.251067	0.134931
350	1.405636	0.872982	0.479759	0.24196	0.130135
360	1.389652	0.850655	0.463107	0.23323	0.125776
370	1.372542	0.827373	0.446921	0.224187	0.120709
380	1.3523	0.801974	0.429353	0.21561	0.115903
390	1.328713	0.775748	0.411958	0.206097	0.110785
400	1.305381	0.750878	0.395754	0.197338	0.106164
410	1.281389	0.72603	0.380124	0.189179	0.102013
420	1.255718	0.701463	0.364934	0.181104	0.098191
430	1.231889	0.679227	0.351692	0.174005	0.094111
440	1.205188	0.656065	0.337728	0.167459	0.090823
450	1.178648	0.634521	0.325107	0.160897	0.087622
460	1.15045	0.612724	0.312898	0.154565	0.084507
470	1.123214	0.59248	0.301584	0.149053	0.081286
480	1.094139	0.572143	0.290286	0.143385	0.078848
490	1.065626	0.552615	0.279669	0.137845	0.075884
500	1.037117	0.533947	0.269231	0.132996	0.073444
510	1.007206	0.514982	0.259254	0.127914	0.071114
520	0.979041	0.497701	0.249895	0.123576	0.068848
530	0.950517	0.480424	0.240789	0.118963	0.06676
540	0.92269	0.463935	0.23209	0.115015	0.064766
550	0.89627	0.448779	0.224208	0.110966	0.062633
560	0.869219	0.433246	0.216099	0.107477	0.061086
570	0.843309	0.418885	0.208782	0.104031	0.059229
580	0.819162	0.405537	0.201926	0.100739	0.057649
590	0.794438	0.392076	0.194927	0.097476	0.056223
600	0.771166	0.379566	0.188426	0.094485	0.054635
610	0.748927	0.367697	0.182513	0.091454	0.053301
620	0.726731	0.35642	0.176775	0.088835	0.051942
630	0.706012	0.345209	0.17134	0.086378	0.050662
640	0.686051	0.334716	0.165838	0.083943	0.049441
650	0.666193	0.324705	0.16083	0.081643	0.048421
660	0.647265	0.314966	0.155845	0.079567	0.047321
670	0.62845	0.305543	0.151089	0.077155	0.046277
680	0.610963	0.296553	0.146537	0.075219	0.04523
690	0.594017	0.288014	0.142521	0.073325	0.043829
700	0.576957	0.279616	0.137977	0.071352	0.043204
710	0.560502	0.272225	0.134564	0.070473	0.042194
720	0.544346	0.262832	0.129886	0.066461	0.040451
730	0.529098	0.255387	0.12591	0.065154	0.039823
740	0.514066	0.248466	0.122591	0.064355	0.039225
750	0.49963	0.241205	0.118983	0.062546	0.038602
760	0.485206	0.23405	0.115469	0.06119	0.037674
770	0.471787	0.227311	0.111921	0.0593	0.037046
780	0.458674	0.220573	0.108307	0.057635	0.036417
790	0.445133	0.214186	0.105149	0.056301	0.035744
800	0.433077	0.208464	0.102521	0.055258	0.035037

Table S6. UV-Vis spectral intensity data for Φ400 nm glass beads.

Φ400 nm	Spectral Intensity [OD]				
Wavelength [nm]	×1	×0.5	×0.25	×0.125	×0.0625
330	1.402104	0.92427	0.513509	0.265267	0.132159
340	1.381888	0.894865	0.493092	0.253863	0.126686
350	1.358876	0.864937	0.472715	0.243043	0.121555
360	1.335026	0.834544	0.453215	0.232659	0.116846
370	1.308502	0.805719	0.435495	0.223835	0.112704
380	1.279743	0.776307	0.417448	0.21457	0.108767
390	1.247797	0.745737	0.399163	0.204932	0.10398
400	1.215218	0.716248	0.381415	0.195405	0.09941
410	1.181022	0.687273	0.364349	0.186362	0.094811
420	1.145917	0.658126	0.347466	0.177252	0.090205
430	1.111911	0.632878	0.332826	0.169883	0.085865
440	1.076414	0.606471	0.317911	0.161944	0.082382
450	1.041895	0.582331	0.304361	0.155163	0.078844
460	1.00704	0.558201	0.291281	0.148143	0.07521
470	0.973965	0.536543	0.279175	0.141989	0.072001
480	0.94076	0.514821	0.2675	0.135816	0.068897
490	0.908394	0.49536	0.256671	0.130308	0.066014
500	0.877476	0.475988	0.246444	0.12482	0.063256
510	0.846455	0.456976	0.235928	0.119857	0.060584
520	0.817436	0.439767	0.226961	0.115222	0.058295
530	0.788712	0.423176	0.218126	0.110491	0.055913
540	0.761523	0.40737	0.209762	0.106098	0.053865
550	0.735454	0.392852	0.201741	0.102414	0.051898
560	0.709804	0.378114	0.194334	0.098685	0.04975
570	0.685153	0.364432	0.18723	0.094949	0.048072
580	0.661742	0.351573	0.180577	0.091393	0.046662
590	0.638889	0.338739	0.173861	0.087997	0.044691
600	0.616876	0.326827	0.167639	0.085095	0.043015
610	0.595859	0.315732	0.161536	0.081947	0.041677
620	0.57536	0.304303	0.155981	0.079309	0.039929
630	0.555843	0.294111	0.150618	0.076535	0.03853
640	0.536815	0.283602	0.145153	0.073709	0.037109
650	0.518469	0.273782	0.140078	0.071246	0.035766
660	0.500838	0.264514	0.135294	0.068733	0.034326
670	0.48398	0.255509	0.130688	0.066347	0.033349
680	0.467994	0.246943	0.12594	0.063985	0.032019
690	0.452358	0.238812	0.122117	0.061955	0.030739
700	0.437267	0.231017	0.117865	0.059972	0.029911
710	0.42323	0.223075	0.113721	0.057911	0.028718
720	0.409177	0.216117	0.11028	0.056271	0.027331
730	0.39644	0.20883	0.106288	0.054023	0.026254
740	0.383906	0.202279	0.102882	0.05251	0.025328
750	0.371818	0.196017	0.099813	0.050841	0.024453
760	0.360371	0.190135	0.096449	0.04926	0.023907
770	0.349501	0.184304	0.093759	0.047578	0.023039
780	0.339211	0.17889	0.090301	0.045992	0.022238
790	0.328997	0.173189	0.087732	0.044585	0.021603
800	0.319106	0.168664	0.08511	0.042986	0.020443

Table S7. UV-Vis spectral intensity data for Φ200 nm glass beads.

Φ200 nm	Spectral Intensity [OD]				
Wavelength [nm]	×1	×0.5	×0.25	×0.125	×0.0625
330	1.493102	0.742136	0.378124	0.192051	0.088596
340	1.402271	0.690977	0.352139	0.178986	0.082047
350	1.317472	0.644722	0.328573	0.167192	0.076187
360	1.234833	0.601798	0.306161	0.15583	0.070414
370	1.165116	0.565177	0.287706	0.146573	0.065998
380	1.096912	0.529953	0.270513	0.13836	0.061719
390	1.031859	0.496983	0.253608	0.129729	0.057541
400	0.972564	0.467224	0.238353	0.122107	0.053656
410	0.917091	0.439416	0.224157	0.114605	0.05009
420	0.862697	0.412828	0.209893	0.107145	0.046577
430	0.817425	0.390377	0.198554	0.101284	0.04385
440	0.770753	0.367673	0.187282	0.095514	0.041005
450	0.728769	0.346893	0.176539	0.090302	0.038511
460	0.687697	0.32703	0.166196	0.084647	0.035819
470	0.651837	0.309782	0.157214	0.08031	0.033744
480	0.616517	0.292819	0.148341	0.075804	0.03151
490	0.584191	0.277222	0.140438	0.07123	0.02968
500	0.552725	0.26241	0.132677	0.067751	0.027774
510	0.522768	0.247875	0.12545	0.063918	0.025886
520	0.495258	0.235179	0.118835	0.06053	0.024625
530	0.469107	0.22213	0.112339	0.057425	0.022794
540	0.445469	0.210644	0.106476	0.054388	0.021552
550	0.42278	0.199784	0.100912	0.051645	0.020067
560	0.4009	0.189233	0.095421	0.048836	0.01885
570	0.380069	0.179601	0.090483	0.045999	0.01761
580	0.361598	0.170514	0.085917	0.043623	0.016613
590	0.342846	0.161759	0.081509	0.041396	0.01551
600	0.326026	0.153796	0.077205	0.039506	0.014632
610	0.309954	0.146185	0.07347	0.03702	0.013795
620	0.294473	0.138673	0.06954	0.035382	0.012964
630	0.279909	0.132265	0.066051	0.033388	0.012127
640	0.26655	0.125645	0.062542	0.031521	0.011449
650	0.253519	0.119244	0.059599	0.030341	0.010814
660	0.241424	0.113375	0.056318	0.028407	0.009973
670	0.23	0.108192	0.053174	0.026854	0.009127
680	0.21887	0.102666	0.0507	0.025651	0.00874
690	0.209086	0.098132	0.048356	0.023972	0.007712
700	0.198799	0.093224	0.045628	0.022667	0.007223
710	0.189316	0.088722	0.043489	0.022058	0.006856
720	0.181528	0.085253	0.041132	0.019584	0.006082
730	0.173239	0.08106	0.03903	0.019441	0.005699
740	0.16539	0.077231	0.037486	0.018898	0.005102
750	0.157802	0.073791	0.035632	0.017635	0.004956
760	0.150721	0.070715	0.033701	0.016797	0.004328
770	0.144628	0.067667	0.032292	0.016076	0.004153
780	0.137911	0.064207	0.03056	0.01528	0.003789
790	0.131717	0.061409	0.029101	0.014597	0.003218
800	0.126148	0.05901	0.027865	0.013778	0.003171

Table S8. UV-Vis spectral intensity data for Φ70 nm glass beads.

**Φ70 nm**	Spectral Intensity [OD]				
Wavelength [nm]	×1	×0.5	×0.25	×0.125	×0.0625
330	1.515195	0.780319	0.400342	0.207015	0.106594
340	1.369148	0.703235	0.361422	0.187336	0.096229
350	1.238035	0.635615	0.327169	0.170041	0.087238
360	1.11846	0.57348	0.295492	0.153619	0.078681
370	1.017329	0.522028	0.269515	0.140587	0.072136
380	0.924042	0.473958	0.246014	0.129153	0.066159
390	0.839566	0.430324	0.223634	0.11754	0.060423
400	0.765931	0.392381	0.20449	0.108124	0.05561
410	0.699075	0.35813	0.18707	0.098914	0.051074
420	0.637092	0.326099	0.17002	0.089884	0.046423
430	0.586084	0.30049	0.156728	0.083135	0.043096
440	0.537073	0.275098	0.144488	0.076994	0.040195
450	0.494307	0.253104	0.132606	0.070924	0.03714
460	0.454146	0.232578	0.121709	0.06532	0.034255
470	0.419984	0.215318	0.113068	0.060844	0.03189
480	0.387927	0.198973	0.104312	0.056297	0.029634
490	0.35944	0.184766	0.09692	0.052191	0.027696
500	0.333014	0.171384	0.089934	0.048905	0.025809
510	0.308677	0.15856	0.083495	0.04551	0.023917
520	0.287047	0.147763	0.077754	0.04286	0.022764
530	0.267096	0.137208	0.072112	0.039909	0.021304
540	0.249373	0.127806	0.067571	0.037554	0.020261
550	0.232747	0.119938	0.063082	0.035252	0.019006
560	0.217407	0.11162	0.059063	0.033411	0.017881
570	0.203235	0.104635	0.055331	0.031226	0.01689
580	0.190762	0.098223	0.051864	0.029507	0.016058
590	0.178854	0.092248	0.048769	0.027979	0.015295
600	0.1678	0.086654	0.045866	0.026496	0.014588
610	0.158158	0.081675	0.042845	0.025001	0.013831
620	0.148717	0.076732	0.04052	0.023562	0.013205
630	0.139689	0.072859	0.038369	0.022131	0.012723
640	0.13194	0.068651	0.036025	0.021249	0.012005
650	0.124522	0.064761	0.033903	0.020483	0.011582
660	0.117584	0.061232	0.031953	0.01931	0.010995
670	0.111507	0.058511	0.029902	0.018321	0.010334
680	0.10509	0.055037	0.028351	0.017509	0.010039
690	0.100057	0.052752	0.027045	0.016438	0.009132
700	0.094518	0.049836	0.025527	0.01596	0.008786
710	0.08952	0.047878	0.02451	0.015506	0.008568
720	0.08536	0.04505	0.022058	0.013001	0.007445
730	0.081099	0.042822	0.021193	0.013237	0.007428
740	0.077239	0.041659	0.020706	0.013436	0.007201
750	0.073615	0.039809	0.019663	0.012791	0.007101
760	0.069907	0.038111	0.018671	0.01252	0.006606
770	0.067146	0.036652	0.017812	0.011959	0.006398
780	0.063725	0.034652	0.016716	0.01138	0.006067
790	0.06092	0.033274	0.015962	0.011125	0.005749
800	0.058148	0.032021	0.015134	0.010384	0.005574

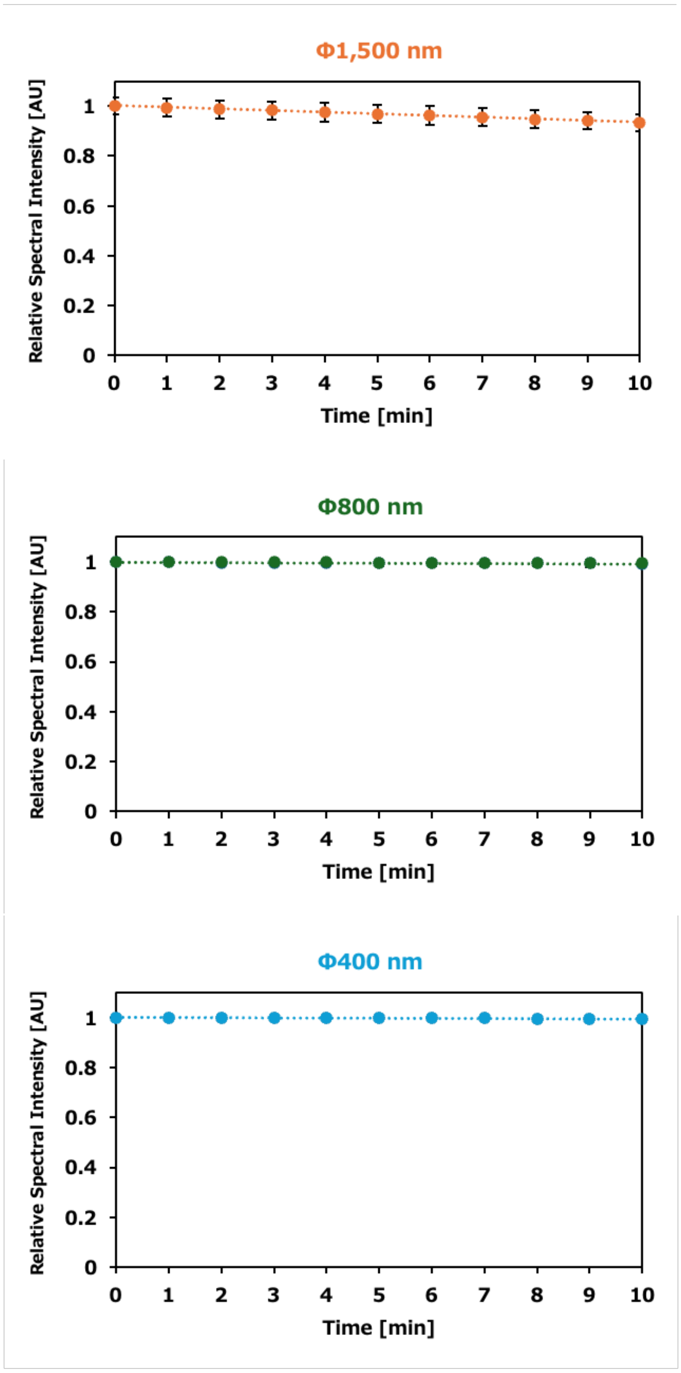

Figure S1. Effect of plate shaking on the relative spectral intensity at 330 nm for glass beads with particle sizes of Φ1,500 nm (top), Φ800 nm (middle), and Φ400 nm (bottom). After shaking at 60 shots per minute for 1 min, the spectral intensity was measured every minute for 10 min. A slight decrease over time was observed for the Φ1,500 nm glass beads.

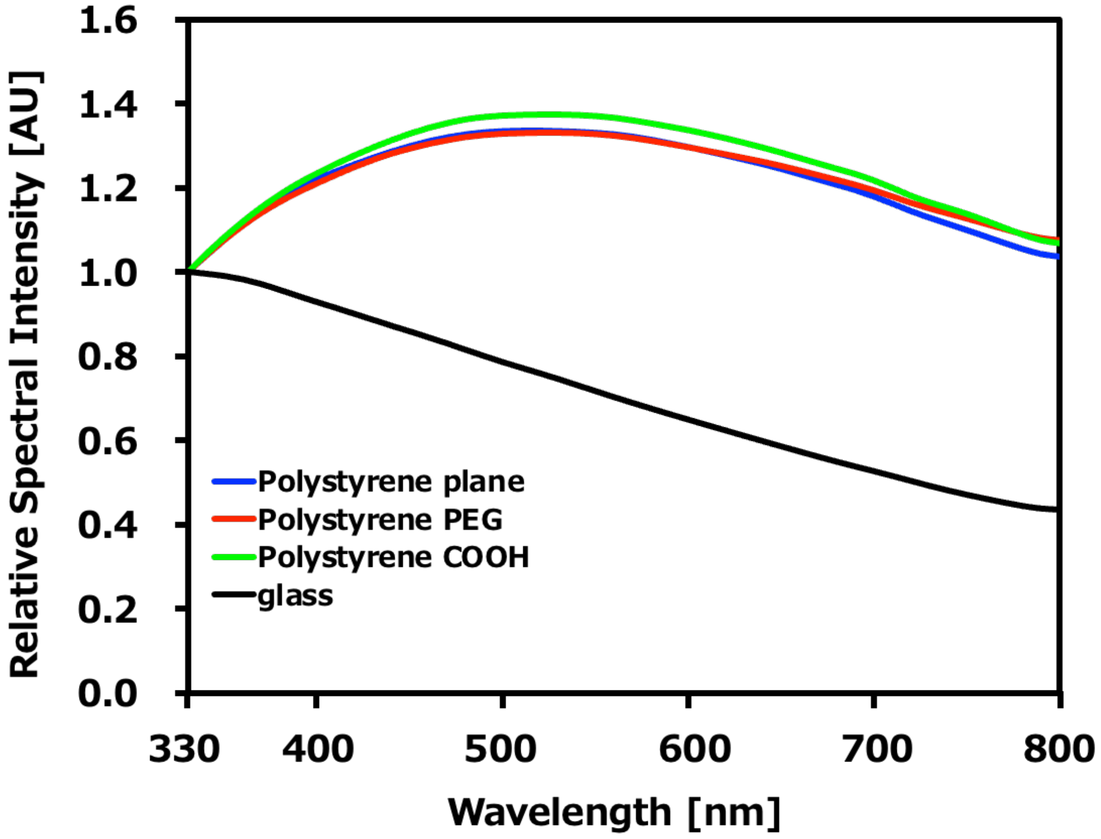

Figure S2. UV-Vis spectra of polystyrene (PS) and glass beads with a particle size of Φ1,000 nm. The spectra of PS-plane (blue), PS-PEG (red), PS-COOH (green), and glass (black) are shown.

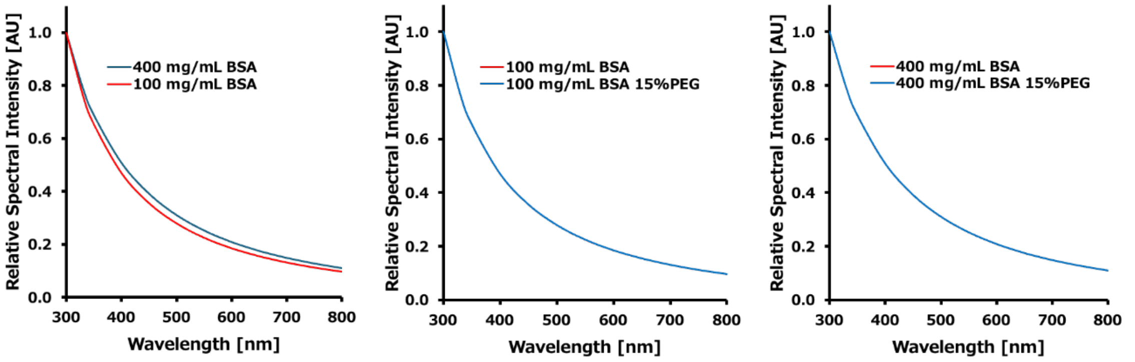

Figure S3. Simulated spectra of BSA-water droplets. (Left) Spectral comparison of droplets with protein concentrations in BSA droplets of 100 mg/mL and 400 mg/mL. (Middle and right) Spectral comparison of simulated spectral shapes of Φ800 nm BSA-water droplets with and without PEG-8000. Protein concentrations in the BSA droplets are 100 mg/mL (middle) and 400 mg/mL (right). PEG-8000 is assumed to be present at a concentration of 15% both inside and outside the droplet.

a

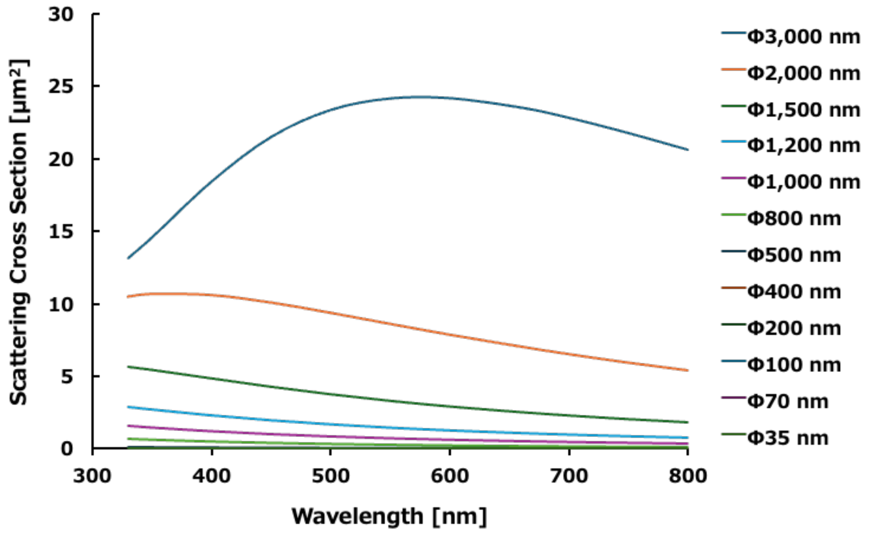

b

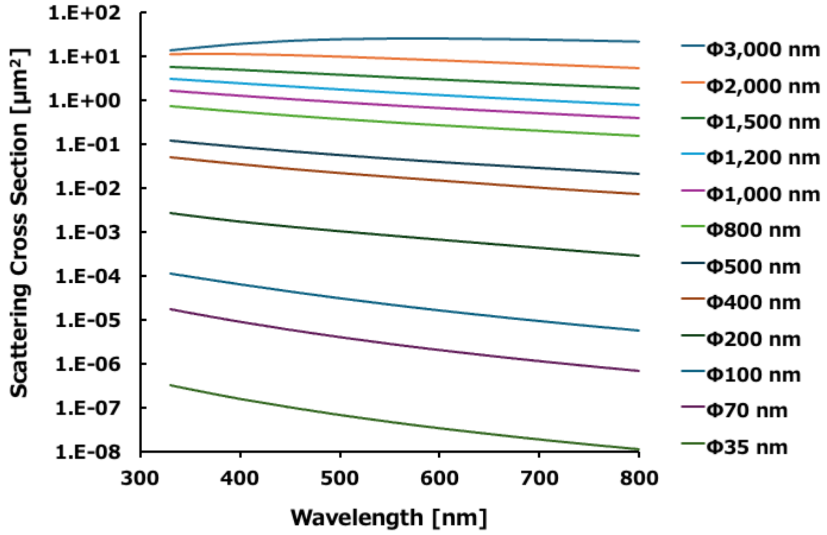

Figure S4. Particle size-dependent changes in the UV-Vis scattering/turbidity spectra. Computer simulations were performed to calculate the spectra of glass beads of various sizes. The shape and absolute intensity of the UV-Vis scattering/turbidity spectrum change with the particle size of the scattering spheres. The vertical axis is the scattering cross section (C_{sca}, μm^2) on a linear (a) and in a logarithmic (b) scale. Note that when the density (concentration) of the scattering spheres is the same, the observed optical density (*OD*) is directly proportional to these quantities.

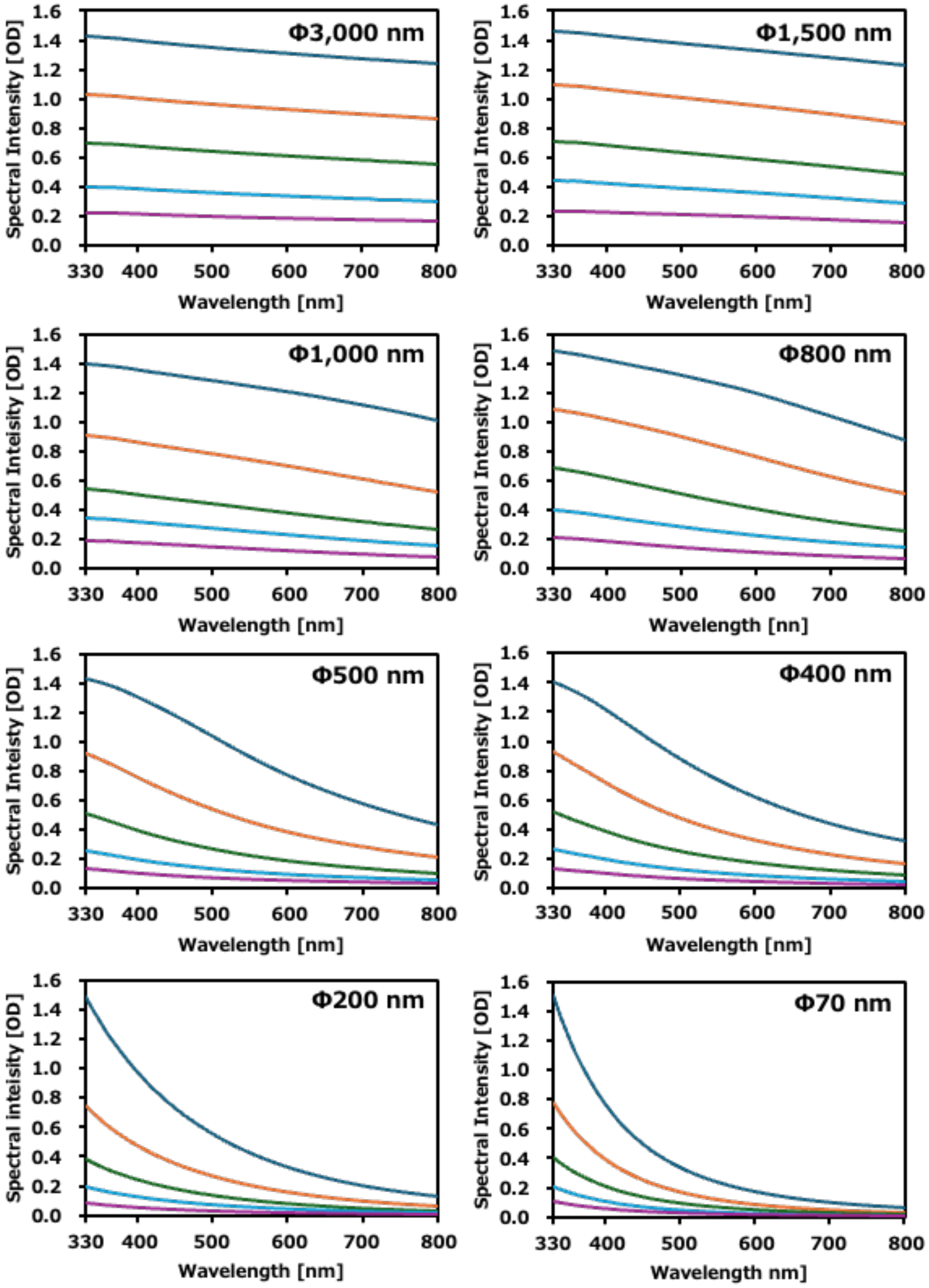

Figure S5. UV-Vis spectra of glass beads with eight different particle sizes (Φ 70, Φ200, Φ400, Φ500, Φ800, Φ1,000, Φ1,500, and Φ3,000 nm). UV-Vis spectra were measured for each sample: a stock suspension (×1, dark blue) with an OD_{330} value of approximately 1.5, as well as its twofold dilutions (×0.5, orange; ×0.25, green; ×0.125, light blue; and ×0.0625, purple).

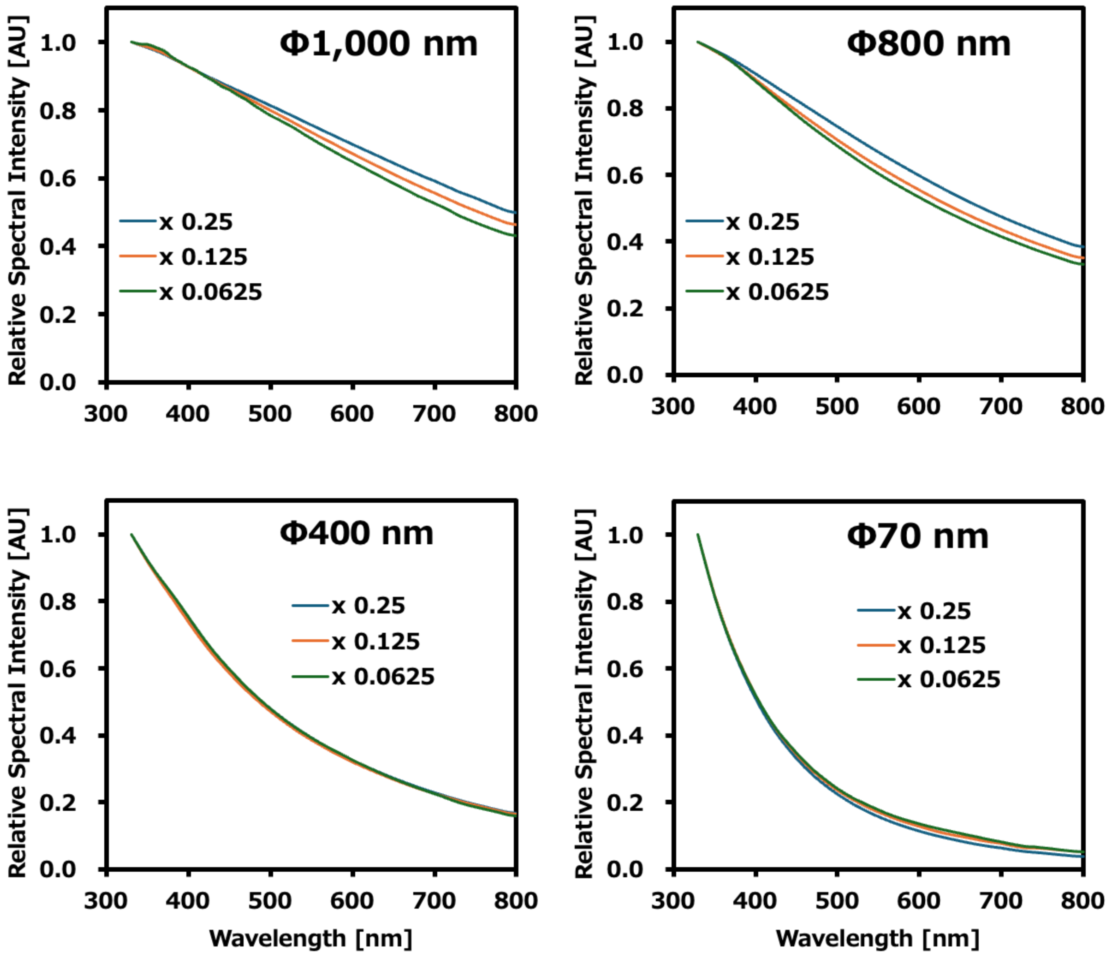

Figure S6. Observed spectral shapes of glass beads of four different particle sizes (Φ70, Φ400, Φ800, and Φ1000 nm) for the OD values below 0.4, corresponding to the dilution factors of ×0.25 (blue), ×0.125 (orange), and ×0.0625 (green). The concentration dependency on the spectral shape is negligible for smaller particles (below Φ400 nm), but not for larger particles (Φ800 nm or larger).

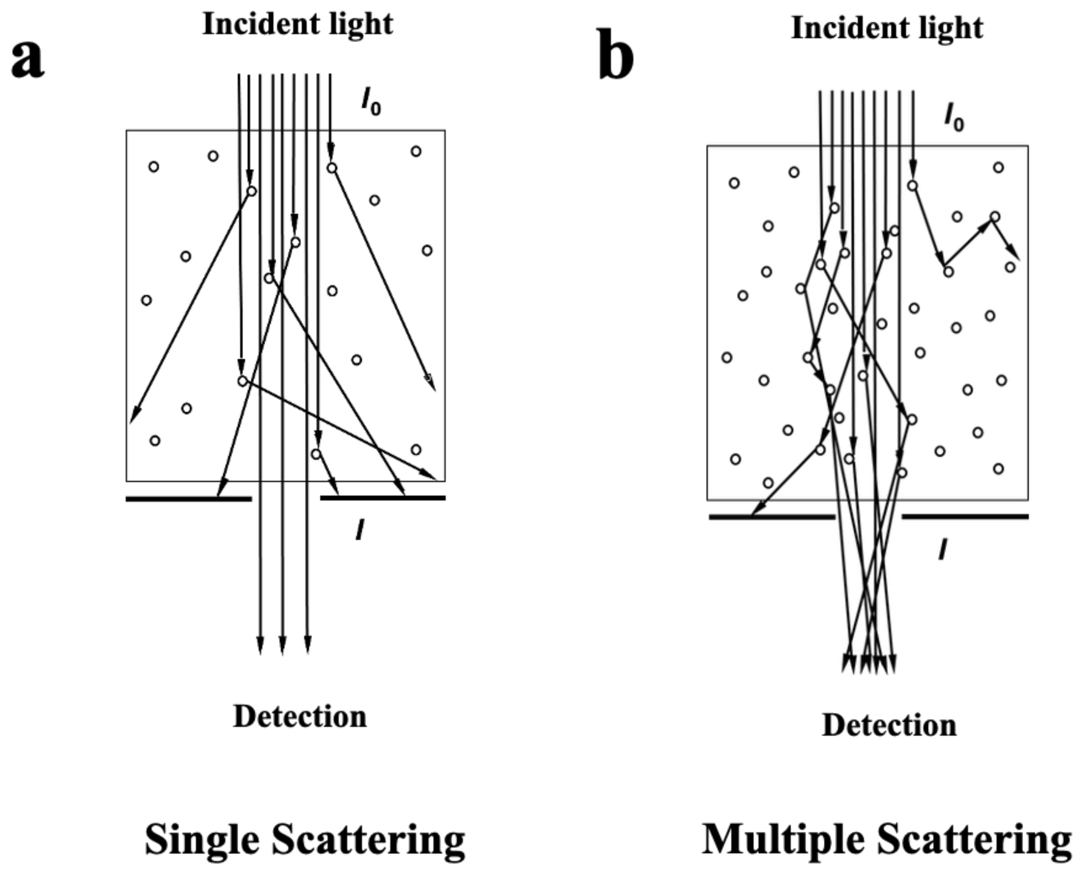

Figure S7. Schematics of incident light disturbance by scattering. (a) Single scattering; (b) multiple scattering. In the latter case, note that the scattered light is scattered multiple times, with a certain percentage detected in the forward direction.

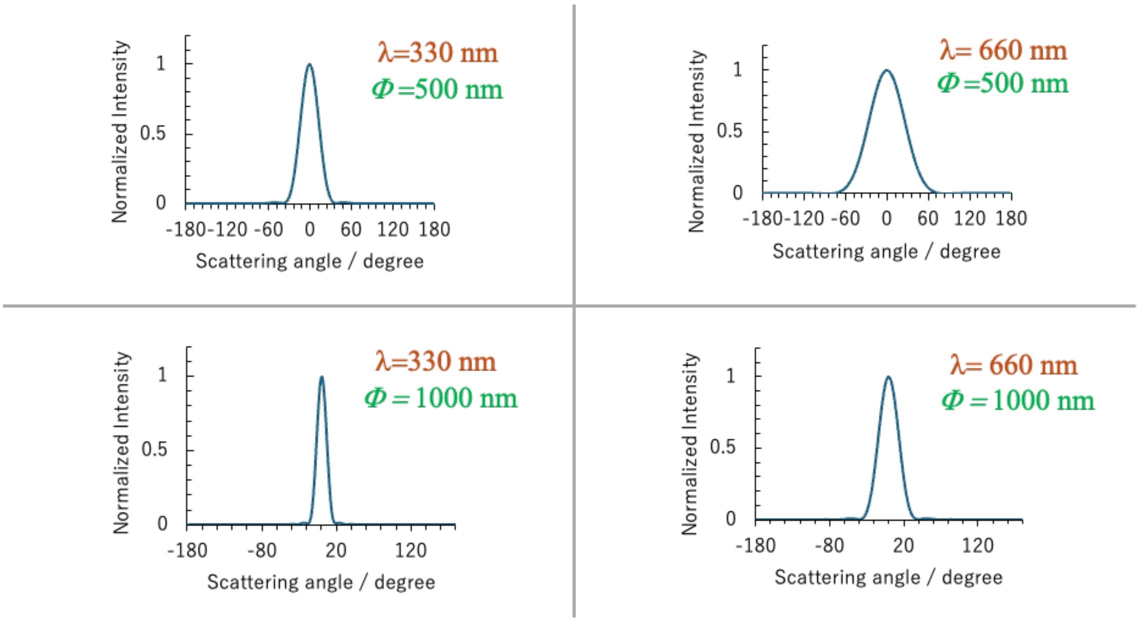

Figure S8. Angular dependence of the scattering intensity (shape of the phase function) for glass beads with diameters of Φ500 nm (top panels) and Φ1,000 nm (bottom panels) at wavelengths of 330 nm (left panels) and 600 nm (right panels). The horizontal axis represents the scattering angle, and the vertical axis represents the scattered light intensity normalized by the strongest intensity. It varies in a complicated manner depending on particle size and wavelength.
